# *Gymnadenia winkeliana*—A New Orchid Species to Romanian Flora

**DOI:** 10.3390/plants13101363

**Published:** 2024-05-14

**Authors:** Nora E. Anghelescu, Lori Balogh, Mihaela Balogh, Nicoleta Kigyossy, Mihaela I. Georgescu, Sorina A. Petra, Florin Toma, Adrian G. Peticila

**Affiliations:** 1 Faculty of Horticulture, University of Agronomic Sciences and Veterinary Medicine of Bucharest, 59 Marasti Blvd, District 1, 011464 Bucharest, Romania; 2 Association “Comori de pe Valea Prahovei”, 106100 Sinaia, Romania

**Keywords:** *Gymnadenia*, *Gymnadenia winkeliana*, micromorphology, morphology, orchids, pollination biology, taxonomy, ultrastructure

## Abstract

A novel species, *Gymnadenia winkeliana*, has been identified in the Bucegi Natural Park ROSCI0013, located in the Southern Carpathians of Central Romania. Two moderately sized populations of *Gymnadenia winkeliana*, totalling 120–140 individuals, were discovered inhabiting the alpine grasslands of the park, situated 2.000 m above sea level. To describe this newly found population as comprehensively as possible, 44 vegetative and floral organs/organ parts were directly studied and measured from living plants. Special attention was focused on the characteristics that proved to have taxonomic significance, particularly those involving distinctive details in the morphology of the leaves, perianth, labellum and gynostemium. A total of 223 characteristics were analysed encompassing the morphology of every organ of the plant, cytology and breeding system. Furthermore, comprehensive taxonomic treatment and description, accompanied by colour photographs illustrating the holotype, are provided. Voucher specimens were deposited at the Herbarium of the University of Agriculture and Veterinary Medicine, Bucharest (USAMVB Herbarium barcode: 40102, NEA); *Gymnadenia winkeliana*, a (micro)endemic species, is characterized as a putative allogamous, facultatively apomict that significantly differs from other *Gymnadenia* R.Br. species found in Romania. Notably, it distinguishes itself through its smaller habitus (reaching heights of up to 8–10 cm), its two-coloured, rounded/hemispherical inflorescence displaying a gradient of pink hues in an acropetal fashion (ranging from whitish-pink at the base to vivid-pink at the topmost flowers), and its limited distribution in high-altitude areas, encompassing approximately 8–10 km^2^ in the central area of the Bucegi Natural Park. This species has been under observation since 2005, with observed population numbers showing a significant increase over time, from ca. 50–55 (counted at the time of its discovery) to 120–140 individuals (counted in June 2023). Additionally, comprehensive information regarding the habitat, ecology, phenology and IUCN conservation assessments of *Gymnadenia winkeliana* are provided, including maps illustrating its distribution.

## 1. Introduction

The genus *Gymnadenia* R.Br. [first published in W.T.Aiton, Hortus Kew. 5: 191 (1813)] is a member of the Subtribe Orchidinae Dressler & Dodson, 1960/Verm., 1955, Tribe Orchideae Dressler & Dodson, 1960/Verm., 1977, Subfamily Orchidoideae Lindl., 1826, Family Orchidaceae Juss., 1789 [1,2].

Etymology: the generic name *Gymnadenia* is a compound term that originates from the ancient Greek words *gymnós-* (meaning naked or bare) and -*aden* (referring to a gland), ad litteram meaning “*with naked glands*”, a reference to the viscidia—the adhesive disks found at the base of the pollinia, also termed *viscidial glands* [3]—that, usually, are free, exposed and not contained within bursicles (pockets of protective tissue), hence the reference “*with naked viscidia*”, used for the orchid species of this genus [3,4,5]. Due to their noticeable sweet fragrance, *Gymnadenia* R.Br. orchids are commonly known as fragrant orchids [1,6,7]. 

**Figure 1:**
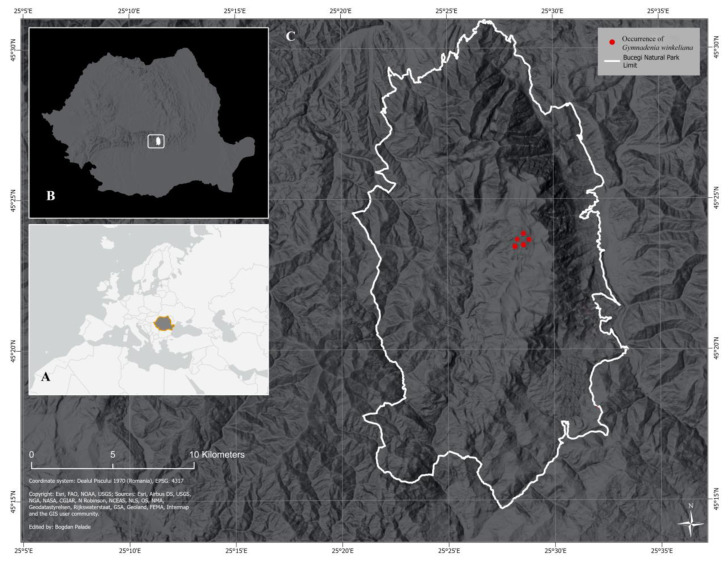
Geographical distribution of *Gymnadenia winkeliana* N.Anghelescu, L.Balogh, M.Balogh & N.Kigyossy. (**A**) Map of Europe, North Africa, the Middle East and Asia; Romania (dark-grey coloured). (**B**) Map of Romania. (**C**) Map of Bucegi Natural Park (BNP) ROSCI001, Southern Carpathians, Central Romania. Known locations of the type specimens (holotype) *Gymnadenia winkeliana*, with an extent of area of occupancy (AOO) of 2.5–5 km^2^ (red dots). Map created by Bogdan Palade [8].

**Figure 2:**
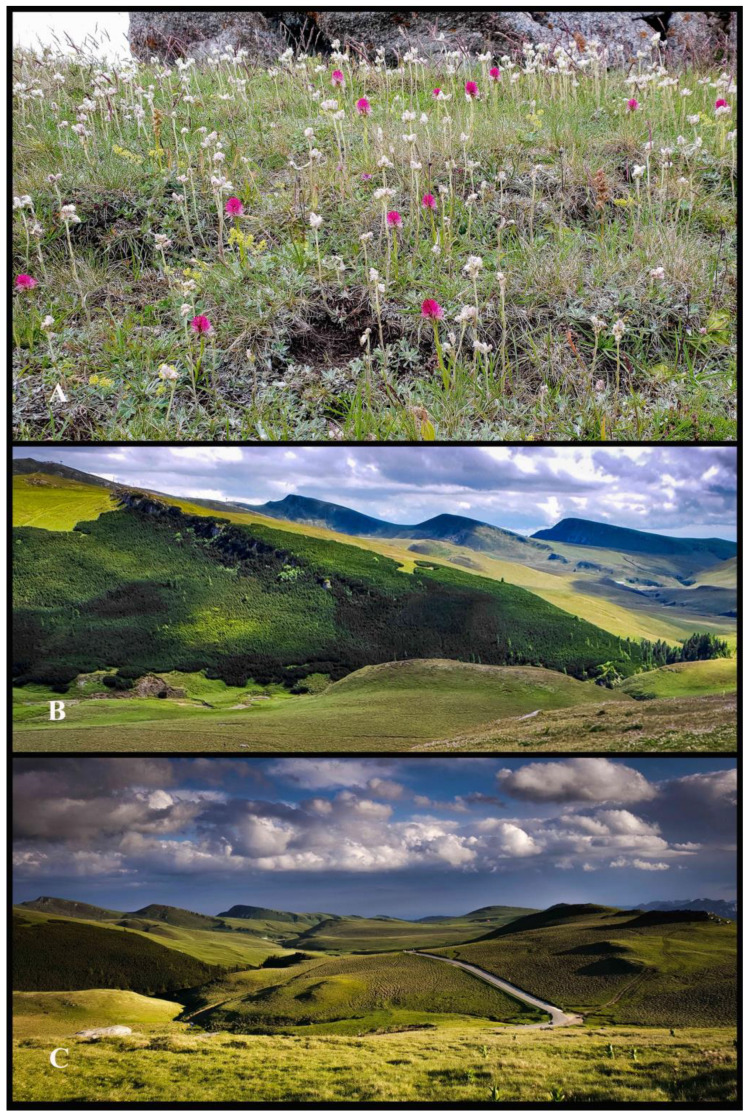
*Gymnadenia winkeliana* N.Anghelescu, L.Balogh, M.Balogh & N.Kigyossy occurring in grasslands in Bucegi Natural Park. (**A**–**C**) *Gymnadenia winkeliana* prefers alpine grassland. The population, counting approximately 120–130(140) individuals, was found to be spread over an alpine plateau (grassland) with an EOO of ca. 2.5–5 km^2^, within the northern-central area of Bucegi Natural Park. (**B**,**C**) The Park is characterized by an unpredictable cold, wet and frequently overcast microclimate, with low temperatures and strong winds. Photographs by Nora E. Anghelescu (**A**) 27 June 2021, (**B**) 2 July 2018, (**C**) 29 June 2022, Bucegi Natural Park (BNP), Romania.

*Gymnadenia* R.Br. aggregate, which encompasses the former genus *Nigritella* Rich., represents a circumpolar genus of terrestrial orchids that extends across temperate Eurasia to Central India. Its range spans from Portugal to Kamchatka, including regions, such as China, Japan, Mongolia, Siberia, the Himalayas and Iran [6,8,9]. Estimates indicate that the total number of species within this aggregate ranges from 15–16 [6,10] to 25–26 [2,11] and 31 [12]. In Romania, *Gymnadenia* R.Br. species occur mainly in alpine regions, of Eastern and Southern Carpathians, with a great diversity in Bucegi Natural Park (Figure 1 and Figure 2).

**Figure 3:**
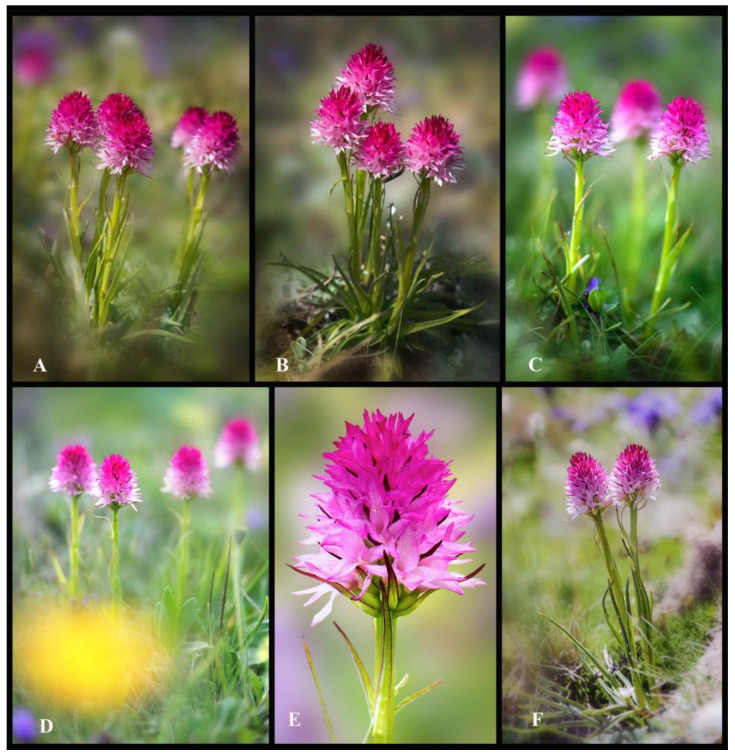
*Gymnadenia winkeliana* N.Anghelescu, L.Balogh, M.Balogh & N.Kigyossy in its natural habitat. (**A**–**F**), *Gymnadenia winkeliana* in its natural habitat. (**A**,**B**,**F**) Compact clumps/clusters of ca. 2–4 *Gymnadenia winkeliana* individuals resulted from vegetative propagation via multiple root tubers produced on the same short rhizome. (**B**) A soldier beetle, *Cantharis obscura* Linnaeus, 1758 (family Cantharidae Imhoff, 1856) is foraging for nectar or floral exudates on the foreground inflorescence. (**C**,**D**) Groups of *Gymnadenia winkeliana* growing in close proximity. (**E**) Detail of the inflorescence showing the swollen basal ovaries, a typical sign of early apomixis onset. Photographs by Nora E. Anghelescu (**A**,**D**) 29 June 2020, (**B**,**C**) 3 July 2023, (**E**,**F**) 28 June 2018, Bucegi Natural Park (BNP), Romania.

**Figure 4:**
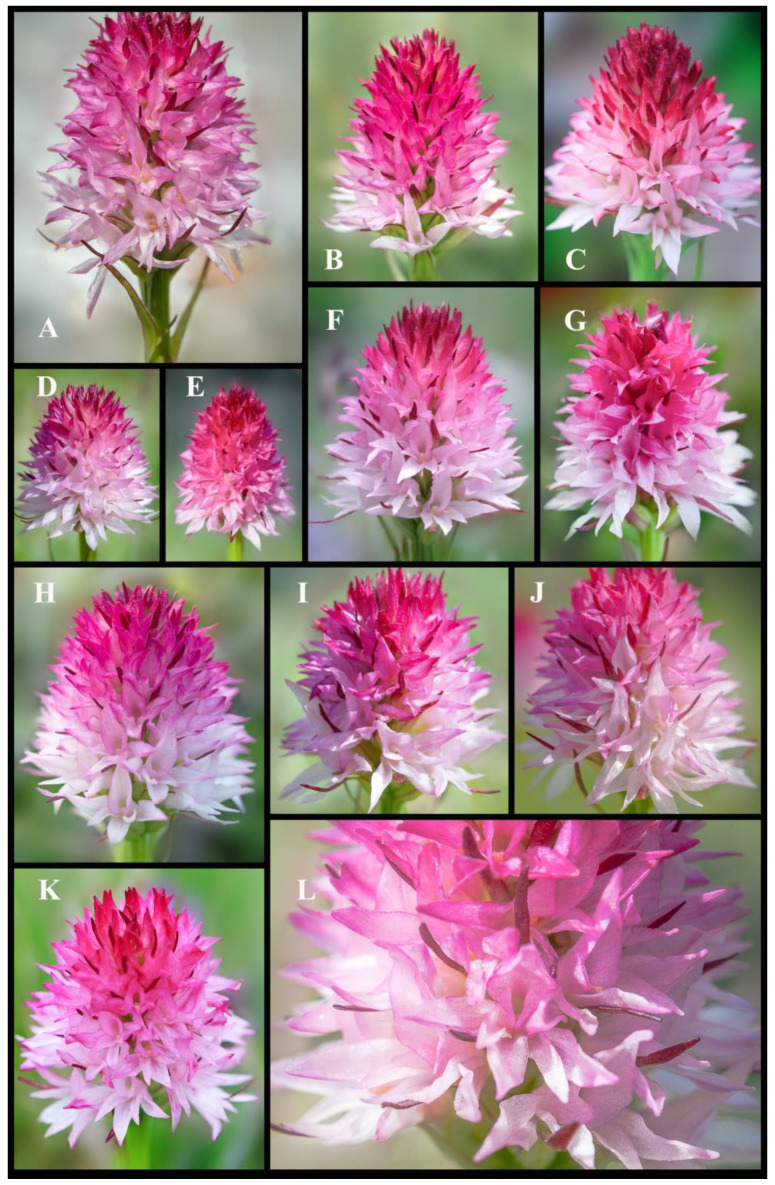
*Gymnadenia winkeliana* N.Anghelescu, L.Balogh, M.Balogh & N.Kigyossy inflorescence details. (**A**–**L**) Details of various inflorescences. The inflorescences are densely packed, near-spherical to subcylindrical racemes (at full anthesis), exhibiting a distinctive gradient of pink hues, with white or pale pink basal flowers and pink to dark pink topmost flowers and buds. The specific colouration is highlighted as a main distinguishing characteristic of the species. (**A**,**B**,**F**–**I**) Inflorescences showing swollen basal ovaries, a typical sign of early apomixis onset. (**L**) Detail of the white-to-pale pink basal flowers of the inflorescences; the flowers are non-resupinate, chasmogamous, with purple-red pigmented bracts, longer than the flowers; on one of the flowers, a pollinarium may be seen, a sign of entomophily/allogamy (insect pollination). Photographs by Nora E. Anghelescu (**A**,**B**) 29 June 2020, (**C**) Nicoleta Kigyossy 29 June 2021, (**D**–**J**) 26—29 June 2021, (**L**) 3 July 2023, BNP, Romania.

**Figure 5:**
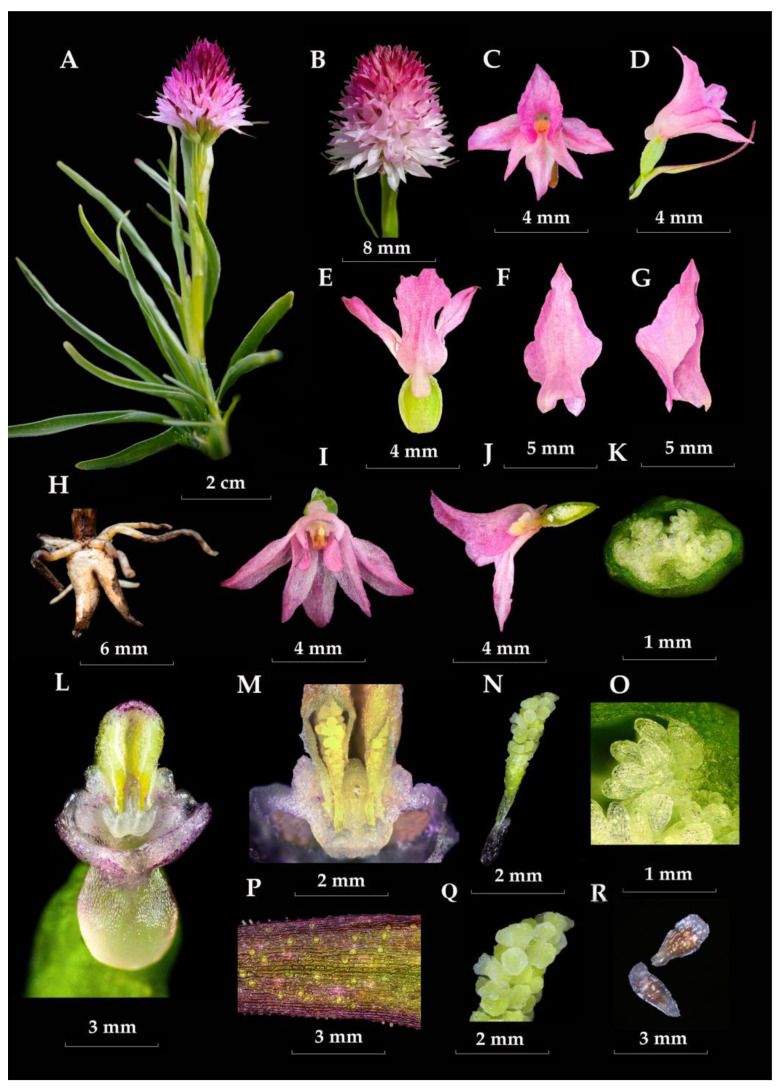
Lankester Composite Dissection Plate (LCDP) of *Gymnadenia winkeliana* N.Anghelescu, L.Balogh, M.Balogh & N.Kigyossy. (**A**) Habitus and leaves. (**B**) Inflorescence exhibiting the characteristic gradient (dégradé) of pink hues. (**C**) Flower—frontal view. (**D**) Bract-ovary-flower unit—side view. (**E**) Ovary-flower unit—above view; swollen ovary indicates apomixis. (**F**) Labellum—abaxial/lower surface view. (**G**) Labellum—adaxial/upper surface view. (**H**) Root tubers (deeply digitate) and adventitious roots formed on a very short rhizome. (**I**) Perianth segments and gynostemium (labellum missing)—frontal view. (**J**) Flower-ovary unit—transversal section side view. (**K**) Ovary—transversal section. (**L**) Gynostemium and spur—frontal view. (**M**) Anther—frontal view (pollinia placed inside the dehiscent anther). (**N**) Massulate pollinarium—pollinia, caudicle and viscidium unit. (**O**) Ovules. (**P**) Bract and anomocytic stomata—upper surface. (**Q**) Massulae—detail. (**R**) Seeds—detail. Illustration and photos by Nora E. Anghelescu from the holotype, 5 July 2023 BNP, Romania.

Although classified as sister genera, the orchid taxa *Gymnadenia* R.Br. and *Nigritella* Rich. are easily distinguishable and are often recognized as distinct genera by many authors, primarily due to morphological evidence [13,14,15,16,17]. Members of *Gymnadenia* R.Br. are characterized by a highly elongate inflorescence (which can reach up to 50 cm in length in certain individuals), with resupinate flowers carrying a slender, medium-sized-to-highly elongated spur, half-filled with abundant nectar [18,19]. In contrast, *Nigritella* Rich. species have a short and compact/dense, head-like inflorescence (measuring 1–2.5 cm in length), very small flowers (Figure 3 and Figure 4), with mild flower colour polymorphism (observed in some species) and non-resupinate flowers carrying a minute sac-like spur (Figure 5), in which much reduced amounts of nectar are secreted [6,14,15,20,21]. However, despite their differences, the two genera share several common traits, including a deeply divided palmate-digitate tuber (Figure 5H), narrow unspotted leaves (Figure 5A) and similar morphologies of the gynostemium [3,6,20].

To date, in Romania, the aggregate *Gymnadenia* R.Br. comprises a modest total of eight taxons, including four diploid (2n = 2x = 40) species, *Gymnadenia conopsea* (L.) R.Br., *Gymnadenia densiflora* (Wahlenb.) A.Dietr., *Gymnadenia odoratissima* (L.) Rich., *Gymnadenia frivaldii* Hampe ex Griseb., and four species of the former genus *Nigritella* Rich., the diploid *Gymnadenia carpatica* (Zapał.) Teppner, E.Klein & Zag. and the tetraploids (2n = 4x = 80) *Gymnadenia miniata* (Crantz) Janch, *Gymnadenia austriaca* (Teppner & E.Klein) P.Delforge and *Nigritella nigra* subsp. *bucegiana* Hedrén, Anghel. & R.Lorenz [1,22,23].

*Gymnadenia* R.Br. orchids are commonly found in alpine and sub-alpine regions, where they prefer habitats with abundant sunlight. They thrive in meadows, grasslands, rocky slopes and other exposed areas, often growing on calcareous/alkaline nutrient-poor (oligotrophic to mesotrophic) substrates (Figure 2A–C). Their habitat extends from altitudes of 1.600 to 2.300 m above sea level (m a.s.l.) [1,22,24,25].

In this paper, we describe and illustrate a new species within the *Gymnadenia* R.Br. aggregate (former genus *Nigritella* Rich.), named *Gymnadenia winkeliana*. The population was first documented in June 2005 by botanist Dan Anghelescu, as part of an orchid field study conducted on the alpine plateau in the central region of Bucegi Natural Park, located in the Southern Carpathian Mountains. The population was found at an altitude of approximately 2000 m above sea level (a.s.l.) (Figure 2B,C).

The main distinguishing features of *Gymnadenia winkeliana*, which immediately attracted his attention, were its small-to-medium habitus, distinct pale-pink colour of the flowers and hemispherical-to-subcylindrical shapes of the inflorescences (Figure 3A–F and Figure 4A–K). These features were highly distinctive and very conserved within the 40–50 individuals discovered.

Since its discovery, the population has been under continuous observation and close monitoring. These key distinctive morphological characteristics of *Gymnadenia winkeliana* have remained consistent over time, with the new individuals displaying little-to-no variation. Over the last eighteen years, the initially rather reduced population more than doubled, indicating that the small, vulnerable plants were thriving and gradually expanding across a larger area.

Nevertheless, the species’ limited area of occurrence and proximity to tourist resorts and cattle farms have been considered as potential significant threats. However, it was likely the confined area of its occurrence that kept it relatively hidden from the widespread human intervention, such as collectors, photographers, tourists and the constant presence of grazing animals often found in Bucegi Natural Park.

Consequently, we chose to formally describe this new taxon as *Gymnadenia winkeliana*, in confidence that further explorations may reveal additional undiscovered populations within the park’s greater area.

Thus, the aims of the present study are as follows: (1) conduct detailed biometrical/morphometrical measurements of both vegetative and floral organs; (2) provide a comprehensive discussion on the key morphological, distinctive characteristics of *Gymnadenia winkeliana*; (3) analyse its potential facultative apomictic origin; (4) conduct a comprehensive study of the pollinator community present within its restricted habitat, documenting its main insect visitors and potential pollinators; (5) identify the most frequent pollinator(s) of this species and discuss the potential facultative allogamy employed by *Gymnadenia winkeliana*; (6) provide information on various aspects of its geographical range, habitat preferences, ecological interactions, phenology and IUCN conservation status, complemented by illustrations and photographs derived from living specimens (the holotype); (7) offer a complete taxonomical treatment of this newly identified species.

Given the significance and rarity of *Gymnadenia winkeliana*, we strongly advocate for its recognition as a new addition to the Romanian flora.

## 2. Results

### 2.1. Area of Occupancy (AOO)–Extent of Occurrence (EOO)

*Gymnadenia winkeliana* is micro-endemic with a restricted distribution, forming a unique population of ca. 120–140 individuals found exclusively in a single location, in the northern-central area of Bucegi Natural Park. Therefore, its Area of Occupancy (AOO–the total area within which a species is known to occur) coincides with its Extent of Occurrence (EOO–the total area encompassing all known locations of a species) (IUCN, 2024). The population was found to be spread over an alpine plateau (grassland) with an AOO/EOO of ca. 2.5–5 km^2^; locus classicus GPS of 45°23′06.82″ N, 25°28′27.81″ E, elev. 1.990–2.010 m a.s.l.; and currently in Dâmbovița County, Bucegi Mountains Natural Park ROSCI001, Southern Carpathians, Romania (Figure 1C, red dots).

### 2.2. Location Studied

Bucegi Natural Park is characterized by an unpredictable microclimate, with low temperatures and strong winds. In the northern-central area, situated at altitudes of 2.500–2.000 m a.s.l., the climate is predominantly cold, wet and frequently overcast (Figure 2B,C). Snow and frost persist for 7–8 months, with an average annual temperature of −3 °C (at Omu Peak, 2.505 m a.s.l.). Rainfall is common, often accompanied by cold temperatures and hail [26]. Conversely, the southern area (2.300–1.800 m a.s.l.) experiences a warmer and drier climate, with more sunshine and less wind and an average annual temperature of 0 °C [23].

### 2.3. Sites Studied

The study sites were located on moderately damp-to-dry, calcareous substrates, at an altitude ranging between 1.990 and 2.010 m above sea level (m a.s.l.). The *Gymnadenia winkeliana* population occurred in full-sun alpine meadows and pasturelands, rich in alpine herbaceous species and visited by a diverse community of pollinators (Figure 1C, indicated by red dots, Figure 2A–C).

### 2.4. Population Studied

A population of approximately 120–130(140) individuals of *Gymnadenia winkeliana* species is included in this study (Figure 2A). The population was found to be spread over an alpine plateau (grassland), on an area measuring approximately 2.5–5 km^2^ currently in the Bucegi Mountains Natural Park (Figure 1C, red dots). The population, initially discovered at the locus classicus in 06.2005, counted ca. 50–55 individuals, a number that increased to 80–100 individuals (2012–2020) and then to ca. 120–130(140), although the number typically remained below 200 individuals (n < 200). The population numbers vary due to the dormancy periods of this species, which typically last one vegetative season from our observations. Furthermore, plants tend to be more numerous and robust in years characterized by abundant snowfalls and rainy springs, rather than in drought years. Moreover, it is possible that the initial population numbers were higher, as the areas frequently used as sheep and cattle fields and a portion of the vegetation may have already been damaged by grazing animals.

### 2.5. Species Studied

*Gymnadenia winkeliana* individuals were studied according to the morphology, habitat, flowering time, geographic location and accessibility. Measurements of the vegetative and floral parts were made from living plants and fresh flowers. To describe this newly found population as comprehensively as possible, 44 vegetative and floral organs/organ parts were directly studied and measured from living plants. Special attention was focused on the characteristics that proved to have taxonomic significance, particularly those involving distinctive details in the morphology of the leaves, perianth, labellum and gynostemium. A total of 223 characteristics scored encompassed the morphology of every organ of the plant, cytology and breeding system. The characteristics (listed in Table 1) described in detail are the roots (8), stem (9), leaves and bracts (36), inflorescence and flower (16), sepals and petals (17), labellum and spur (30), gynostemium (13), anther (13), pollinia and pollinarium (27), stigma (19), ovary (12), fruit (6), seed and embryo (15), chromosomes (2), flowering time and reproductive strategies (7). For laboratory stereomicroscope biometrical measurements, several flower-bract units were extracted from a position located approximately one-third to halfway from the base of the inflorescence. Only flowering parts (individual flowers and 1–2 inflorescences) of the plants were sampled, leaving the vegetative parts for persistence and continued growth.

### 2.6. Morphometric/Biometric Data

**Figure 6:**
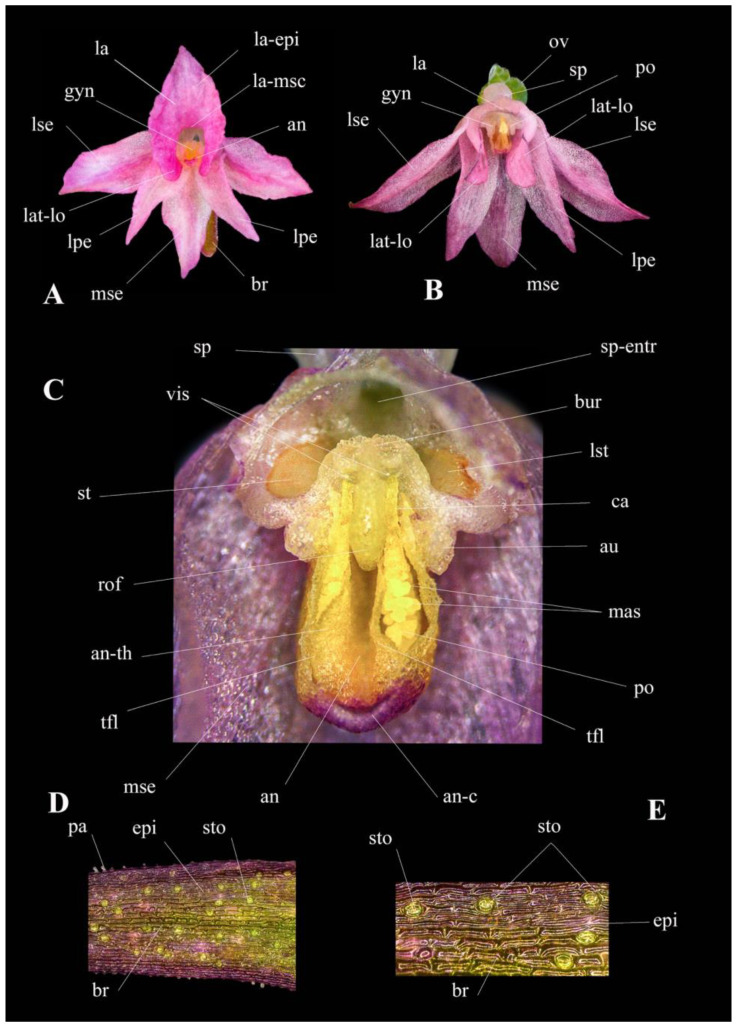
*Gymnadenia winkeliana* N.Anghelescu, L.Balogh, M.Balogh & N.Kigyossy flower structure, and flower section (the labellum was removed) showing the reproductive organs (gynostemium) in the upper, deep-pink flowers. (**A**) Flower—frontal view of the perianth segments and bract; flowers are non-resupinate, and the labellum (epichile) is oriented upwards; labelar lateral lobes form a tunnel (mesochile), which leads to the gynostemium/anther (facing downwards). (**B**) Transversal section of the flower (labellum removed); one pollinarium protrudes off the anther; the spur is short and saccate. (**C**) Gymostemium—detail in its downward (natural) position; stigmatic lateral lobes spread sideways, forming a prominent/protruding rostellar flap; thecae are dehiscent and pollinia are freed from thecae flaps; viscidia are partially contained in a rudimentary, membranous bursicle; the anther connective is purple-pigmented. (**D**,**E**) Bract detail—upper surface presenting anomocytic stomata. Abbreviations: an-c—anther connective; an-th—anther theca; an—anther; au—auricle; br—bract; bur—bursicle; ca—caudicle; epi—epichile; epd—epidermis; gyn—gynostemium; la-msc—labellum mesochile; la—labellum; lat-lo—lateral labellar lobe; lpe—lateral petal; lse—lateral sepal; lsl—lateral stigmatic lobe; mas—massula/massulae; mse—median sepal; po—pollinia; rof—rostellar flap; ros—rostellum; sp-entr—spur entrance; sp—spur; st—stigma; sto—stomata; tfl—thecal flaps; vis—viscidium. Illustration and photos by Nora E. Anghelescu from the holotype, 5 July 2023 BNP, Ro.

**Figure 7:**
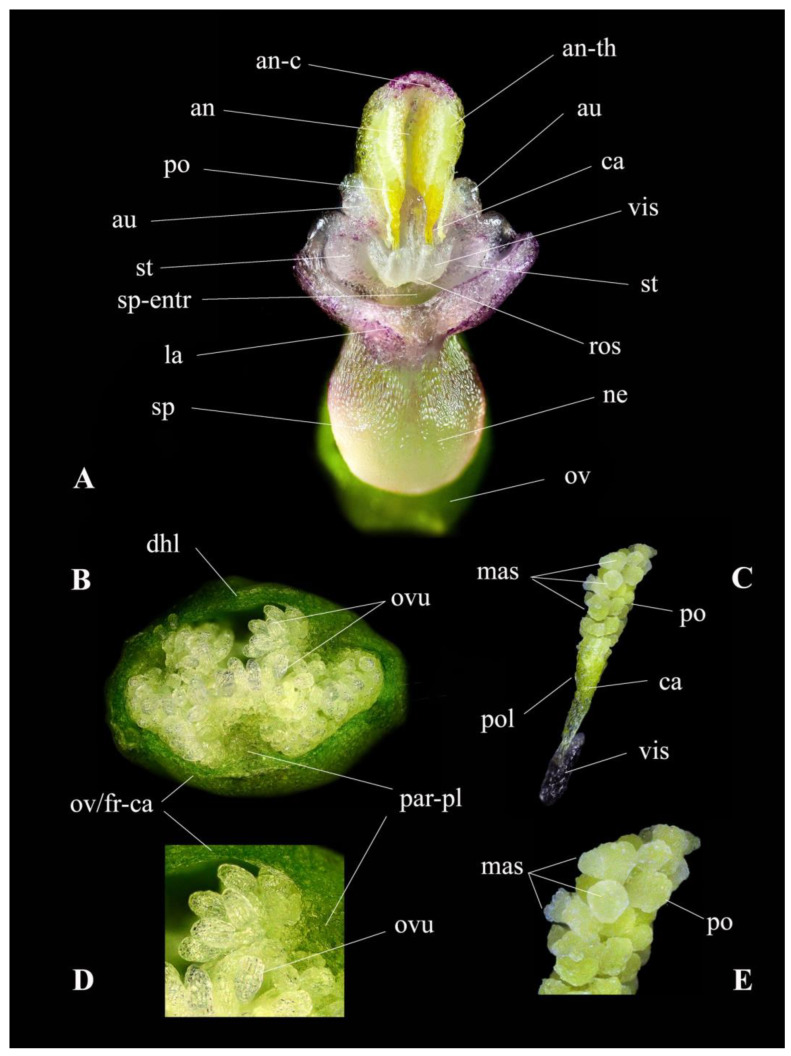
*Gymnadenia winkeliana* N.Anghelescu, L.Balogh, M.Balogh & N.Kigyossy gynostemium-spur unit detail, ovary section and pollinarium structure (section of the young, basal, whitish-pink flowers) (**A**) Gynostemium-spur unit detail in basal (whitish) flowers; purple pigmentation is reduced, showing a reduced concentration of anthocyanins; viscidia are completely enclosed within the rudimentary bursicles; the spur is roundish-spherical, nectar secreting. (**B**) Ovary—transversal section showing parietal placentation; developing ovules are reaching the immature seed stage, showing an already reticulated testa. (**C**) Pollinarium—massulate pollinia-caudicle-viscidium unit; massulae are loosely forming the pollinia; free viscidial discs are translucent and well-developed. (**D**) Ovules—detail of the reticulated testa in ovaries of young, basal flowers. (**E**) Massulae—details. Abbreviations: an-c—anther connective; an-th—anther theca; an—anther; au—auricle; bur—bursicle; ca—caudicle; dhl—dehiscence lines; fr—fruit; mas—massula/massulae; ne—nectar; ov—ovary; ov/fr-ca—ovary/fruit capsule; ovu—ovule; pa-pl—parietal placentation; po—pollinia; pol—pollinarium; ros—rostellum; sp-entr—spur entrance; sp—spur; st—stigma; vis—viscidium. Illustration and photos by Nora E. Anghelescu from the holotype, 5 July 2023 BNP, Romania.

In the light of so much contradictory DNA-based evidence [29], detailed morphological measurements continue to be the primary method for plant identification. Considering the phenotypic plasticity and variations within the genus, it is essential to thoroughly evaluate the macro- and micromorphological characteristics that can be utilized for taxon delimitation. A comprehensive study of the key morphological characteristics, pollinator, flowering phenology, chromosomes and reproductive strategy of *Gymnadenia winkeliana* is listed in Table 1 and illustrated in detail in Figure 3, Figure 4, Figure 5, Figure 6 and Figure 7.

### 2.7. Sympatric Orchid Species

Several other orchid species were recorded to occur sympatrically with *Gymnadenia winkeliana*, having overlapping antheses and sharing a large pollinator community characteristic for their specific alpine habitat. These include *Dactylorhiza viridis* (L.) R.M.Bateman, Pridgeon & M.W.Chase (Figure 2A), *Gymnadenia conopsea* (L.) R.Br., *Pseudorchis albida* (L.) Á.Löve & D.Löve and *Pseudorchis albida* subsp. *tricuspis* (Beck) E.Klein [1,22,23,30].

### 2.8. Pollination Monitoring

**Figure 8:**
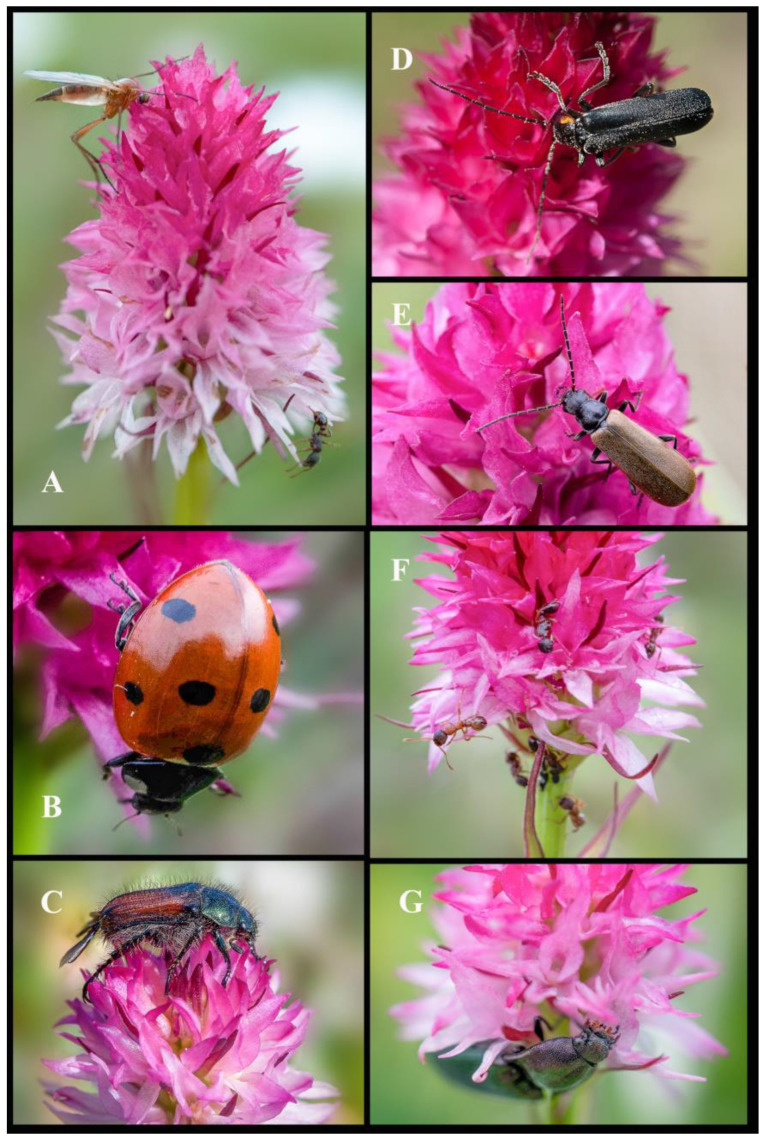
*Gymnadenia winkeliana* N.Anghelescu, L.Balogh, M.Balogh & N.Kigyossy—Coleopteran, Dipteran and Hymenopteran pollinators and visitors. (**A**) *Empis trigramma* (Empididae Latreille, 1804), yellow dance fly, potential true pollinator. (**B**) *Coccinella septempunctata* (Coccinellidae Latreille, 1807), seven-spot ladybird, visitor, carnivorous and feeding on aphids. (**C**) *Phyllopertha horticola* (Scarabaeidae Latreille, 1802), garden chafer, garden foliage beetle, visitor, phytophagous. (**D**) *Cantharis obscura* (Cantharidae Imhoff, 1856), soldier beetle, potential/accidental pollinator, visitor, pollen forager. (**E**) *Rhagonycha lignose* (Cantharidae Imhoff, 1856), golden soldier beetle, visitor, pollen forager. (**F**) *Myrmica rubra* (Formicidae Latreille, 1809), common red ant, European fire ant, accidental pollinator, visitor, carnivorous. (**G**) *Ctenicera cuprea* (Elateridae Leach, 1815), common click beetle, accidental pollinator, visitor, phytophagous. Illustration and photos by Nora E. Anghelescu, June–July 2017–2023 Bucegi Natural Park (BNP), Romania.

**Figure 9:**
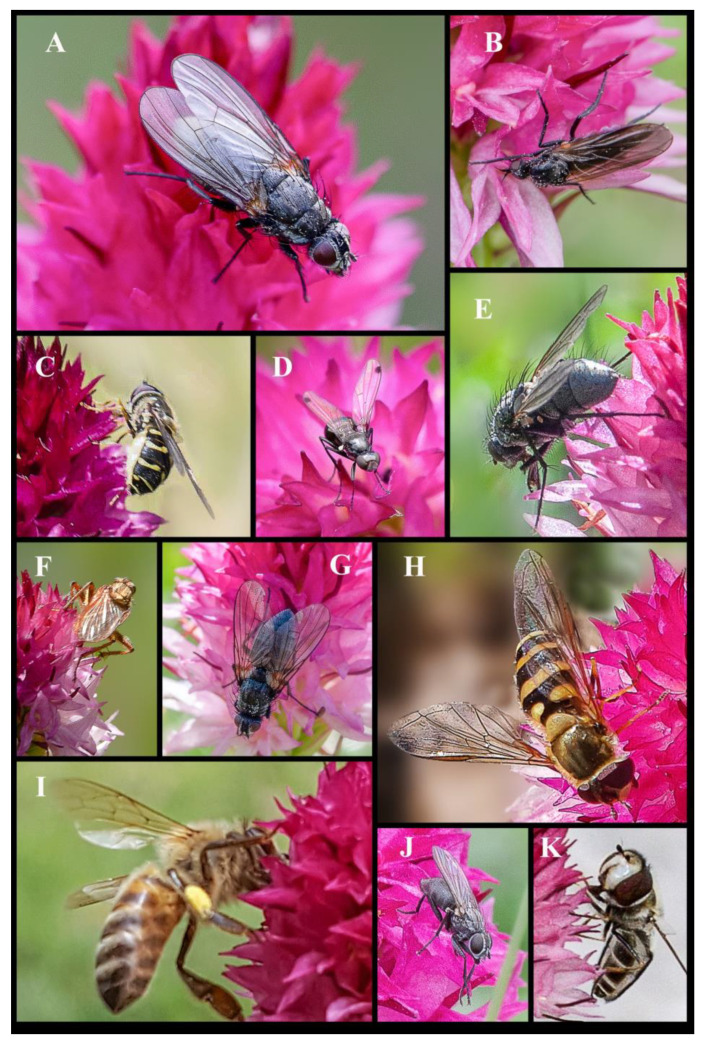
*Gymnadenia winkeliana* N.Anghelescu, L.Balogh, M.Balogh & N.Kigyossy—Dipteran pollinators and visitors. (**A**) *Adia cinerella* (Anthomyiidae Robineau-Desvoidy, 1830), flower fly, potential/accidental pollinator. (**B**) *Empis ciliata* (Empididae Latreille, 1804), black dance fly, true pollinator. (**C**) *Syrphus ribesii* (Syrphidae Latreille, 1802), common hoverfly, accidental pollinator. (**D**) *Sepsis fulgens* (Sepsidae Walker, 1833), lesser dung fly. (**E**) *Calliphora* (cf.) (Calliphoridae Brauer & Bergenstamm, 1889), blow fly, accidental pollinator. (**F**) *Scathophaga stercoraria* (Scathophagidae Robineau-Desvoidy), yellow dung fly, accidental pollinator, visitor. (**G**) *Hydrotaea* sp. (Muscidae Latreille, 1802), house fly, dump fly, accidental pollinator. (**H**) *Syrphus ribesii* (Syrphidae Latreille, 1802), common hoverfly, accidental/potential pollinator. (**I**) *Apis mellifera* (Apidae Latreille, 1802), European honey bee, true pollinator. (**J**) *Pegomya* sp. (Anthomyiidae Robineau-Desvoidy, 1830), beet leafminer, accidental pollinator. (**K**) *Scaeva pyrastri* (Syrphidae Latreille, 1802), Pied hoverfly, accidental pollinator. Illustration and photos by Nora E. Anghelescu, June–July 2017–2023 Bucegi Natural Park (BNP), Romania.

**Figure 10:**
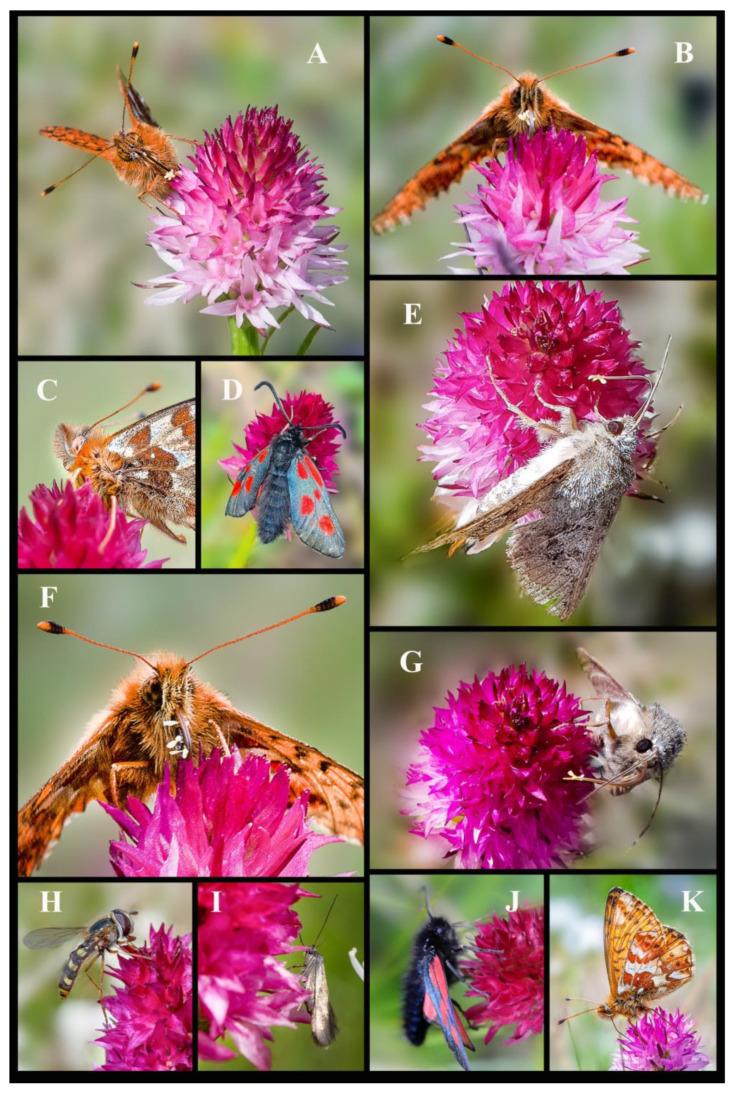
*Gymnadenia winkeliana* N.Anghelescu, L.Balogh, M.Balogh & N.Kigyossy—Lepidopteran and Dipteran pollinators and visitors. (**A**–**C**,**F,K**) *Boloria pales* (Nymphalidae Rafinesque, 1815), shepherd’s fritillary true pollinator. (**D**) *Zygaena exulans* (Zygaenidae Latreille, 1809), Scotch burnet true pollinator. (**E**,**G**) *Pharmacis carna* (Hepialidae Stephens, 1829), ghost moths, furry moth true pollinator. (**H**) *Eupeodes corollae* (Syrphidae Latreille, 1802), common hoverfly, accidental/potential pollinator. (**I**) *Adela* sp. (Adelidae Bruand, 1851), fairy moths, potential pollinator. (**J**) *Zygaena loti* (Zygaenidae Latreille, 1809), slender Scotch burnet true pollinator. Illustration and photos by Nora E. Anghelescu, June—July 2017—2023 BNP, Romania.

Since *Gymnadenia winkeliana* is restricted to its locus classicus, all insect monitoring was conducted exclusively within the above AOO (Area of Occupancy). During our pollination surveys, conducted over several years, 2017–2023, and spanning the months of June and July, when the flowers are in full antheses; we documented a diverse range of insect pollinators/visitors, members of the Orders Coleoptera (beetles), Diptera (flies, mosquitoes), Hymenoptera (ants, bees, wasps) and Lepidoptera (moths, butterflies), all part of the Class Insecta (Figure 8, Figure 9 and Figure 10).

In total, we documented 23 families comprising 47 species of insects that were recorded pollinating and visiting the inflorescences of *Gymnadenia winkeliana*. The total number of pollinator/visitor insect species of each Order is summarised below: (I) Coleoptera—Accidental pollinators: 5 families, 7 species; (II) Diptera—Pollinators (efficient) and potential pollinators: 7 families 21 species; (III) Hymenoptera—Pollinators and potential pollinators: 2 families, 6 species; (IV) Lepidoptera—Pollinators (highly efficient): 9 families, 13 species. Of the species included in this study, approximately 15% were Coleoptera, 44.6% were Diptera, 12.76% were Hymenoptera and 27.65% were Lepidoptera (Figure 8, Figure 9 and Figure 10).

## 3. Discussion

### 3.1. Morphological Characterization of Gymnadenia winkeliana

The main criteria used in the identification of *Gymnadenia winkeliana* included (1) the shape of the inflorescence and flowers, (2) the shape and form of the labellum, (3) the shape and form of the gynostemium, (4) the type of lower bract margins (entire, papillate, serrate, etc.) and (5) the presence or absence of denticles on bracts. These characteristics are important for distinguishing *Gymnadenia winkeliana* from other orchid species and are commonly used by botanists and taxonomists for the accurate identification of species in the field [3,31]. The presence or absence of denticles on bracts was often used as a diagnostic characteristic [4,32,33,34,35]. Nevertheless, while denticulation of bracts has been traditionally used as a diagnostic characteristic for identification, its reliability can vary, leading some authors to question its usefulness as a qualitative characteristic [3,5]. Klein [24] also emphasized the importance of flower colouration as a taxonomically relevant characteristic for some of the species of the subgenus *Nigritella* Rich., stressing its consistency across species. With few exceptions ([e.g., *Gymnadenia rhellicani* (Teppner & E. Klein) Teppner & E. Klein, which shows a wide variety of nuances [5,7]), flower colour is often a more reliable and consistent characteristic than denticulation, making it a valuable tool for the identification of these alpine orchids [10,11,18].

The main morphological characteristics studied are listed below. Their potential taxonomical value is discussed.

***Habitus and Stem*.** Similar to other species of the subgenus *Nigritella* Rich., *Gymnadenia winkeliana* is a perennial that remains dormant as an underground tuber in the winter and produces a single short stem, at the beginning of the vegetative season. The conserved short stature of *Gymnadenia winkeliana*, typically reaching heights of about 8.5–10.5 cm, with occasional specimens reaching up to 14 cm, serves as a distinctive trait. This characteristic helps differentiate it from other orchid species with taller or more variable growth habits. The flowering stems are characteristically erect, slender, flexuous (may aid in adaptation to windy or harsh environmental conditions in the alpine habitat), ridged, glabrous (lacking hairs, trichomes or glandular, epidermal structures) and entirely vivid-green, with no purple pigmentation at the upper parts (absence of anthocyanin pigments). The vivid-green coloration of the stems, without any purple pigmentation at the upper parts due to the absence of anthocyanin pigments, is a distinctive characteristic, aiding in its accurate identification and differentiation from other orchid species (Figure 3A–E).

***Basal leaves*.** *Gymnadenia winkeliana* is a summer-green orchid, i.e., its leaves emerge at the beginning of the vegetative season in spring (typically in May or later if snow persists at higher altitudes) and remain green throughout the summer. Their numbers vary, ranging from 6 to 10(12), and are arranged in a basal rosette. The variation in the number of leaves provides some degree of variability within the population but likely follows a consistent pattern within individuals. The leaves are grass-like, narrowly lanceolate with acuminate tips. The deeply keeled structure and spreading angle relative to the stem (approximately 40°–45°) are likely adaptations for minimizing water loss in alpine habitats. In larger leaves, a central vein and faint parallel venation (multiple ribs) may be observed. The leaves are entirely green with a vivid green upper side and a yellowish-green underside and lack any purple markings (maculae). The leaf surface is smooth, glossy and deep green. The margins are yellowish-green, uniformly smooth, entire and straight, with no undulations present. These characteristics may help differentiate *Gymnadenia winkeliana* from other orchid species with different leaf textures and margin shapes (Figure 5A).

***Cauline leaves***. The 4–7(8) cauline leaves are arranged alternately or in two vertical rows (distichous) evenly spaced along the stem. Unlike the basal leaves, they are triangular-lanceolate sheaths, erect to slightly arched and relatively rigid. They are vivid green with dark-red-to-purple-brown edges and tips, indicating the presence of anthocyanin pigments in the marginal epidermal cells. Their surface is textured, displaying a central vein (midrib). The leaf margins are serrated (presenting an irregular margin) or edged with fine, hyaline, translucent, conical-elongate, tooth-like papillae. The papillae are closely spaced and irregularly distributed, with some leaves having denser coverage than others. The irregular distribution of papillae along the leaf margins, with some leaves having denser coverage than others, suggests variability within the population (Figure 3A–C,F and Figure 5A).

***Bracts.*** The flower bracts are shorter than the cauline leaves but longer than the flowers. They are narrowly triangular and have acuminate tips. The basal bracts spread horizontally at an angle of approximately 90° relative to the stem (perpendicular to the stem), while the median and top bracts are angled at approximately 40°–45° relative to the inflorescence rachis. The bracts exhibit a consistent greenish-brown coloration, with pronounced purple pigmentation at the tips and margins. The margins are finely serrated by numerous, elongate, translucent papillae (denticles), which are typically evenly spaced. While they measure between 0.04 and 0.14(0.28) mm in size, they show moderate variability and create an irregular or serrated margin (irregularly denticulate) (Figure 3E, Figure 4A, Figure 5D and Figure 6D,E).

***Stomata.*** Like all species in the subgenus *Nigritella* Rich., *Gymnadenia winkeliana* bears hypoamphistomatic leaves, with stomata present on both sides of the leaf, predominantly on the lower, abaxial surface [36]. The adaxial surface of the bracts presents numerous anomocytic stomata (a stomatal type in which the subsidiary cells surrounding a stomate are not differentiated from the other epidermal cells [37]), restricted to the apical area [36]. On the adaxial surface (upper surface) of the bract, stomata are restricted to the apical area, towards the tapering, strongly purple-pigmented tip (Figure 5P and Figure 6D,E).

***Inflorescences*.** The inflorescences are densely packed racemes (the flowers are arranged along a central axis, with each flower attached by a short stalk or pedicel), typically carrying an average of 60 to 80 flowers (floriferous). The inflorescences undergo mild changes in shape and size throughout their developmental stages. They start as pyramidal during bud stages, then become near-spherical to hemispherical (subcylindrical) at full anthesis (when flowers are fully open), and finally become ovate to subovate during the fruiting stages. Nevertheless, the size and shape slightly vary among individuals and populations. While there is slight variation in the inflorescence size and shape among individuals, these features are consistent across the species. The inflorescences exhibit a distinctive two-coloured appearance, with a gradient of pink hues. The basal flowers are white or pale pink, while the topmost flowers and buds display a range of shades from pale pink to dark pink. The light pink coloration of the inflorescences is highlighted as a key/main characteristic of the species, serving as a distinguishing feature from other *Gymnadenia* R.Br. species in Romania (Figure 3A–F, Figure 4A–L and Figure 5A–E).

***Flowering pattern*.** The flowers open sequentially from the base upwards in an acropetal manner, meaning that the lower flowers open first, followed by those above them, until reaching the topmost flowers and buds. The inflorescences are very floriferous, bearing 40–60(80) small, pinkish flowers (Figure 4L and Figure 5A).

***Individual Flower*.** Similar to the inflorescences, individual flowers exhibit a colour gradient ranging from white to whitish-pink at the base, transitioning to pink and deep-pink towards the tip of the inflorescence. This gradient creates a visually striking display of colour variation within the inflorescences, which helps in species identification in the field. While the general colour pattern follows a consistent gradient, there is slight variation among individuals, with some displaying a more pronounced pinkish hue at the base. The flowers are wide open (chasmogamous), star-shaped, with spreading petals, which enhances their attractiveness to pollinators. Similar to all other species in the subgenus *Nigritella* Rich., the flowers of *Gymnadenia winkeliana* are non-resupinate (untwisted, i.e., flowers maintain their original orientation throughout development, without undergoing a twisting or rotational movement) (Figure 4L, Figure 5C–E and Figure 6A,B).

***Scent.*** The flowers emit a distinctly sweet, vanilla-like fragrance. This fragrance is likely produced by volatile compounds released by the flowers (which include vanillin and vanillyl ethyl ether), which are perceived by pollinators [6]. We noticed that the fragrance is particularly intense during the morning hours/first part of the day, indicating that scent production may be influenced by environmental factors, such as temperature and humidity. Morning hours are often a peak activity time for many pollinators, so the timing of scent emission aligns with pollinator foraging behaviour. The sweet and vanilla-like fragrance is likely an adaptation to attract potential pollinators. Due to their remarkable chocolate and/or vanilla fragrance, the species in the subgenus *Nigritella* Rich. are known as the European Vanilla Orchids or simply the Vanilla Orchids [1,16,23].

***Flowering time*.** The flowering time typically spans from late June to mid-July, based on field observations conducted from 2004 to 2023. Our field observations indicate that the plants often tend to synchronize their blooming, flowering simultaneously. The majority of flowering occurs during the hottest part of the summer season, typically in June and July, with the longest daylight hours in temperate Romania (Figure 2A, Figure 3A–F and Figure 4A–L).

***Flower longevity*.** The anthesis typically lasts for a period of 4 to 7 days, with some flowers remaining open for up to 10 days. The longevity of the flowers is greatly influenced by environmental factors, such as temperature, rainfall, wind and others.

***Perianth segments*.** The perianth segments, sepals and lateral petals spread laterally, have acute/acuminated, arched tips and exhibit similar colouration that may vary depending on their position within the inflorescence. The sepals are slightly longer and approximately twice as wide at the base as the lateral petals. The median sepal is broader and more pronounced, oriented downward due to the non-resupination of the flower. This positioning may serve as a landing platform for potential insect pollinators or visitors, facilitating pollination interactions (Figure 5C–G,I,J and Figure 6A,B).

***Labellum*.** It is non-resupinate, hence its upward orientation, narrower rhomboidal in shape, with an acuminate tip. It is shallowly three-lobed, consisting of a central lobe and two lateral lobes, with a bulbous base containing the gynostemium. The central lobe (the epichile) is elongate and triangular, with slightly undulating, smooth margins and no markings (spots or dots) present on its surface. The lateral lobes are scalloped and rounded, but less pronounced than the central lobe. The middle segment of the labellum (the mesochile) is saddle-shaped and forms a narrow tube-like junction, approximately 0.6–0.7 mm wide, created by the incurved, narrowing edges of the lateral lobes. In some cases, these edges almost touch each other. The narrowing tunnel leads to the centre of the flower where the reproductive structure, the gynostemium, is situated. The basal part of the labellum (the hypochile) is broad, bulbous and rounded, terminating in a saccate, short spur (Figure 5F,G).

***Spur*.** The spur is nectariferous, translucent-white, straight (not curved or arched) and filled up to ¼–½ of its length with nectar. The presence of nectar in the spur serves as an attractant to pollinators, potentially contributing to the reproductive success of the orchid. Additionally, the shape and size of the spur, along with other floral characteristics, such as the labellum, have been frequently used as discriminatory features in the identification of various species within the genus *Nigritella* Rich. [33,34,38,39,40] (Figure 5D,E,I,L and Figure 7A).

***Gynostemium***. In Orchidaceae, the reproductive structures—the unique, fertile anther and the stigma—are fused into a singular, columnar central structure known as the gynostemium. In *Gymnadenia winkeliana*, the gynostemium is thick, cylindrical and translucent in appearance. The gynostemium is positioned horizontally or slightly facing downwards, with the stigma located above the anther due to the non-resupination characteristic of the flower, above or adjacent to the entrance of the spur. The downwards (non-resupinated) orientation of the gynostemium, which places the stigmatic cavity above the anther, makes self-pollination impossible, a main characteristic of all species of the subgenus *Nigritella* Rich. Laterally, the gynostemium features two verrucose gynostemial auricles (Figure 5I,J,L,M, Figure 6B,C and Figure 7A).

***Anther*.** In *Gymnadenia winkeliana*, the anther is bithecal, containing two separate/individual, parallel, elongated, translucent white thecae (loculi/chambers). They are connected by a well-developed, translucent white-to-yellowish connective tissue. The connective tissue often has a purple-pigmented roof, indicating the presence of anthocyanins (Figure 5L,M, Figure 6C and Figure 7A).

***Pollinia*.** Each theca contains an ovoid-elongate, yellow, massulate (granular) pollinium, formed by individual densely packed massulae. The massulae are ovoid blocks of pollen grains (tetrads) held together by elastoviscin [41,42]. At the terminal end, the pollinia form a thin, long, translucent-yellow structure called the caudicle that typically measures more than 30% of the length of the pollinium. This caudicle serves as a connecting structure between the pollinium and the viscidial disk. The viscidial disk is a circular, adhesive structure located at the end of the caudicle. It allows the pollinarium to adhere to the proboscides of potential pollinators, aiding in pollen transfer during pollination [41] (Figure 5M,N,R, Figure 6C and Figure 7A,C,E).

***Pollinarium*.** The pollen dispersal unit (PDU), also known as a pollinarium, is composed of a massulate pollinium, a caudicle, and a free viscidial disk [41,42,43]. Unlike other species of orchids, the viscidial disk in *Gymnadenia winkeliana* is enclosed or contained within a rudimentary membranous bursicle. In older flowers, it becomes more exposed and freer to adhere to potential pollinators. There are two pollinaria per anther, one in each theca, containing all the pollen of the flower. All species of the genus *Gymnadenia* R.Br. possess massulate pollinia/pollinaria as part of their reproductive structures [44]. Additionally, in some species, the viscidia may be contained in rudimentary bursicles [5,11] (Figure 5M,N, Figure 6C and Figure 7C).

***Stigma*.** Due to the non-resupinated feature of the flower, the stigma is located above the anther (the gynostemum points downwards). The placement of the stigma above the anther makes spontaneous self-pollination impossible [5,41]. The stigmatic cavity is concave elliptical in shape, translucent white and covered with stigmatic exudate, indicating a wet stigma. In massulate orchids, like *Gymnadenia* R.Br. species, the stigma typically consists of three lobes: one central (rostellum) and two lateral lobes. The lateral lobes are broad, flap-like structures that spread laterally [9,45,46] (Figure 5L,M, Figure 6C and Figure 7A).

***Rostellum*.** The rostellum functions as a barrier between the anther, which contains the pollinia pair, and the stigma, preventing even more the occurrence of (accidental) self-pollination in the non-resupinate *Nigritella* Rich. species. The median lobe, also referred to as the rostellar flap, is prominent and protrudes between the two thecae containing the pollinaria, making self-pollination impossible [45]. The well-defined separation created by the highly developed rostellar fold indicates that *Gymnadenia winkeliana* relies on insect pollinators (entomophilous) for cross-pollination, making it a potentially facultative allogamous species (Figure 5L,M, Figure 6C and Figure 7A).

***Ovary*.** The ovary is ovoidal, unilocular/monocarpellar (consisting of a single chamber, lacking the septum) and syncarpous (composed of fused carpels). The ovary is epigynous, meaning it is enclosed within the receptacle with the floral parts arising above it [47]. The ovary is sessile, lacking a flower pedicel, and does not twist since *Gymnadenia winkeliana* has non-resupinate flowers. Placentation is parietal, meaning that the placenta develops along the fused margins of adjacent carpels on the parietal wall of the ovary [48]. In the majority of the plants observed, the basal ovaries were significantly swollen, still having the unwithered/still fresh, anthetic flowers hanging, a putative indication of a species characterized by an early onset of apomixis [47] (Figure 5E,J,K,O and Figure 7B).

***Ovules*.** The ovules are translucent-white to whitish-green, anatropous (inverted during development by the bending of the funicle (stalk) attaching it to the carpel wall) and tenuinucellate (the nucellus is reduced to one layer of cells, which surrounds the megaspore mother cell), with one or two integuments [47] (Figure 5O and Figure 7B,D).

***Fruit*.** The indehiscent fruit is an elongate-ovoidal green pod, characterized by three highly pronounced longitudinal ridges (three lines of dehiscence). Fruiting occurs in June–July, with a relatively high fruit set rate, estimated at approximately 40–68(80)%, based on 45 counts (Figure 3E and Figure 5J).

***Seed capsule***. Seed maturation typically occurs between July and August, with the last capsules dehiscing in September to October. The seed capsule is brownish, elongate-ovoidal and slightly larger than the fruit pod.

***Seeds*.** The seeds are very small (microseeds) and numerous (microspermy), elongated-ovoidal to fusiform in shape, tapering from the middle to the tips, up to twice as long as wide [17] (Figure 5R).

***Testa*.** The seeds are covered in a brownish reticulated testa. The testal cells are rectangular-elongated, with non-sinuous anticlinal walls. The testa is open at the micropyllar end and closed at the chalazal end (micropyle—the opening at the apex of the ovule where the integument does not completely cover the nucellus and allows the pollen tube to enter the ovule; chalaza—the basal part of the ovule where the integuments and nucellus are attached [47] (Figure 5R).

***Embryo*.** Centrally, the seeds contain a spherical-ovoidal rudimentary, whitish-yellow, endospermless embryo. The endosperm in orchid seeds is nuclear (reduced) and non-functional, as it either fails to develop/form or degenerates after the fertilization of the polar nuclei [49] (Figure 5R).

### 3.2. Cytogenetics

The chromosome numbers within the *Gymnadenia* R.Br. genus are known to vary significantly, between diploidy and polyploidy (tri-, tetra- and pentaploidy), with a basic chromosome number of x = 20 [19,31]. Due to the early swelling of the ovaries (a potential sign of apomixis), *Gymnadenia winkeliana* has been suggested to be a polyploid species, more specifically putatively tetraploid, with a chromosome count of 2n = 4x = 80 (polyploidy is usually associated with apomixis). Further research is required to confirm the exact chromosome number of *Gymnadenia winkeliana*.

### 3.3. Reproductive Biology

#### 3.3.1. Asexual Reproduction

##### Vegetative Propagation

*Gymnadenia winkeliana* may have the potential for asexual reproduction through vegetative propagation via root tubers. This is evidenced by numerous compact clusters of 2–4(6) adjacent plants emerging from the same rhizome or multiple root tubers, a phenomenon previously reported by [13,16,44,45]. Although vegetative reproduction is seemingly uncommon, the production of extra tubers can result in clumps of (genetically identical) flowering stems adjacent to each other in some individuals, as shown (Figure 3A,B,F). *Gymnadenia winkeliana* seems to employ, rather often, vegetative propagation since clusters of 2–4 plants were rather often encountered within the population.

##### Apomixis

All members of the subgenus *Nigritella* Rich. have non-resupinate flowers. Consequently, the gynostemium is slightly downward-oriented, positioning the stigmatic cavity directly above the anther, thereby preventing self-pollination. Furthermore, these species possess a well-developed stigmatic median lobe, known as the rostellum, which acts as an additional barrier against accidental self-pollination. As a result, due to the absence of self-pollination, the polyploid *Nigritella* Rich. species employed apomixis.

Our observations showed that, in the majority of flowering individuals, the young basal blooms (at the very beginning of anthesis) presented already significantly swollen ovaries. A transversal section through the basal, young flowers’ ovaries revealed ovules in advanced stages of development, already developing into immature seeds with a distinctly reticulate testa (Figure 7B,D). Nevertheless, we cannot conclude with certainty whether the observed swollen ovaries in *Gymnadenia winkeliana* are the result of apomixis. Still, considering the limited presence of pollinators, we hypothesize that the high occurrence of fruiting ovaries in young plants might suggest the early onset of apomixis. This assumption is based on the fact that, similar to all other species within the subgenus *Nigritella* Rich., self-pollination is not possible in *Gymnadenia winkeliana*. This is due to two main factors: (1) non-resupination, which positions the anther above the stigmatic cavity, and (2) the significant development of the rostellum, which acts as a barrier preventing any contact between the pollinia and the stigmatic cavity.

Apomixis is a form of asexual reproduction that results in the production of seeds without the occurrence of meiosis or fertilization [50,51,52]. As a consequence, through this alternative reproductive mode, clonal seeds (genetically identical to the mother plants) are produced [53].

Apomixis can be categorized into two types based on the origin of the embryo: gametophytic apomixis and sporophytic apomixis (adventitious embryo) [51,54].

In the former genus *Nigritella* Rich., apomixis was shown to be sporophytic and exclusively observed in polyploid species [13,16,34,47,55]. While diploid *Nigritella* Rich. species reproduce sexually (allogamy), their polyploid counterparts reproduce asexually through apomixis [56,57]. Although the association between apomixis and polyploidy was observed several times, [24,34,40], apomixis has only been confirmed in the former genus *Nigritella* Rich. within the subtribe Orchidinae (subfamily Orchidoideae) [13]. Consequently, while the presence of a diploid *Gymnadenia winkeliana* cannot be ruled out, given the connection between apomixis and polyploidy, it is plausible to consider that *Gymnadenia winkeliana* might be a polyploid member of the genus.

In sporophytic apomixis, also known as adventitious embryony, embryos develop directly from the somatic tissues of the ovules, located outside the embryo sac, i.e., from either the nucellar (nucellar embryony) or the integumental cells (integumentary embryony) of the ovule [53,55,58,59]. Additionally, in sporophytic apomixis, the formation of the embryo sac was reported to proceed normally, with the megaspore mother cell (MMC) undergoing multiple meiotic and mitotic cycles, resulting in the development of a seven-celled, eight-nucleate/octanucleate embryo sac [47,60,61]. As a result, embryo sac formation is not directly associated with apomixis, leading to the production of a functional haploid egg cell [58,62]. One of the first types of adventitious (nucellar) embryony has been described by Afzelius (1932) [63] in the tetraploid *Gymnadenia nigra* (L.) Rchb.f., in which simultaneously with the development of the female gametophyte (arrested at the nuclear stage), one or two nucellar embryos were also formed [47].

***Facultative apomixis.***

Similarly to the description given by Afzelius (1932) [63] in *Gymnadenia nigra* (L.) Rchb.f., in most apomictic plants, both sexual and asexual reproduction processes occur simultaneously within the same ovule, a phenomenon referred to as facultative apomixis [61,64,65]. Consequently, for allogamous species (cross-pollinated, entomophilous) (i.e., the fragrant, nectar-rewarding *Gymnadenia winkeliana*, a potential allogamous species), the presence of adventitious embryony, may not entirely prevent the possibility of subsequent pollination events [62].

To date, in the Orchidaceae family, facultative apomixis was mentioned in about 18 genera, i.e., *Cephalanthera* Rich., *Cynorkis* Thouars, *Dactylorhiza* Neck. ex Nevski [66], *Epidendrum* L., *Epipactis* Zinn, *Epipogium* Borkh. [67], *Gastrodia* R.Br., *Genoplesium* R.Br., *Goodyera* R.r. [47], *Gymnadenia* R.Br. (including subgenus *Nigritella* Rich.), *Hammarbya* (L.) Kuntze [47], *Maxillaria* Ruiz & Pav., *Neottia* Guett., Orchis Tourn. ex L., *Rhomboda* Lindl. [68], *Spiranthes* Rich. [69], *Zeuxine* Lindl. [47] and *Zygopetalum* Hook [70].

Facultative apomixis combined with sexual reproduction was demonstrated to occur in the tetraploid (2n = 4x = 80) apomict *Gymnadenia austriaca* (Teppner & E.Klein) P.Delforge [34]. In a cross-pollination experiment with the diploid allogamous *Gymnadenia corneliana* (Beauverd) Teppner & E.Klein (2n = 2x = 40), among the many apomict embryos, two triploid zygots were observed, resulting from the sexual fusion between two haploid egg cells from *Gymnadenia austriaca* (Teppner & E.Klein) P.Delforge (n = ca. 40) and normal haploid sperm cells from *Gymnadenia corneliana* (Beauverd) Teppner & E.Klein (with n = 20), with the two resulting sexual zygots having a chromosome number of ca. 2n = 3x = 60. This experiment clearly shows that functional haploid egg cells can be produced in polyploid apomicts and the subsequent pollination events may result in the successful production of sexually formed zygots, thus increasing the genetic heterogeneity in predominately apomictic populations [71,72].

***Do apomicts still need pollinators?***

Once formed within the ovule, the adventitious embryos engage in competition with the sexual embryo for nutrients [55,59]. In order for their development to progress further, it has been demonstrated that their survival significantly depends on the double fertilization of the sexual embryo sac [52,64,73]. This process not only generates the zygote and the triploid nucleus (resulting in reduced endosperm, in Orchidaceae) but also triggers the developmental start of the ovule [73,74,75]. This initiation is facilitated by complex nutrient and growth signals originating from both the egg cell and sperm cells [58,65,76]. Therefore, in most facultative apomicts, the formation of the embryo sac may still require the presence of pollen on the stigma to initiate the process of embryo sac development [77]. In orchids, in general, microsporogenesis proceeds normally, and the male sexual function is maintained not only for double and triple fertilisation, but also because pollination is essential for ovule development [75,78]. The reliance on pollination for ovule and embryo development might serve as an additional, yet underexplored, constraint influencing the scarcity of apomixis in orchids, which is a relatively rare phenomenon in the family [65,79].

The requirement for pollination in facultative apomixis may explain why facultative apomicts, i.e., *Gymnadenia winkeliana*, a putative apomict, still exhibit all the characteristics needed for allogamy (pollination by insects), such as showy flowers, strong fragrances (vanilla, chocolate), abundant nectar and well-developed pollinaria [19,31]. Therefore, despite being regarded as a potential facultative apomict, *Gymnadenia winkeliana* may still rely, to some extent, on pollinators for the development and maturation of its fruits and seeds, whether they are formed through sexual or asexual reproduction pathways [80,81].

***Colonising Unfamiliar Habitats.***

Although apomixis is rare in Orchidaceae, it has been recognized as a strategy that could potentially provide reproductive assurance independently from pollinators or pollen vectors [68,77,82]. Therefore, apomixis has been thought to be a major advantage for species colonizing areas that were previously glaciated or located at high altitudes [74,83,84]. In such environments, a limited number of highly fit, recently formed/evolved genotypes may rapidly spread over larger areas, particularly at elevated altitudes [83,85,86].

The micro-endemic *Gymnadenia winkeliana* represents a remarkable example of a potential facultative apomict that has successfully colonized a high-altitude harsh habitat characterized by a cold, rainy microclimate, with short, stormy summers, and with a limited presence of pollinators (Figure 2B,C). The significant increase in population numbers reinforces its potential facultative apomictic nature, which, combined with its remarkable adaptability, allowed it to colonize new, unfamiliar ecological niches.

#### 3.3.2. Sexual Reproduction

As discussed, apomixis and sexuality are not mutually exclusive traits, as almost all apomictic plants exhibit facultative sexuality, i.e., allogamy or insect pollination [74,81,83]. *Gymnadenia winkeliana* shares similarities with several other allogamous European species of its genus, at the same time, retaining all the morphological features of a sexual reproductive species. Its brightly coloured chasmogamous flowers, the pleasant vanilla sweet scent (long-distance attractant for pollinators) and the nectar secreted in its saccate spur (reward for potential pollinators) are the main characteristics of an entomophilous species, making this species a good candidate for allogamy. Additionally, its well-developed, functional pollinia and putative sporophytic type of apomixis may enable cross-pollination. Consequently, *Gymnadenia winkeliana* may also be considered a putative facultative allogamous (cross-pollinated) species, relying on entomophily (insect pollination) to produce fruit and seeds sexually, thus reassuring its genetic diversity within its constantly/gradually increasing population. Autogamy is impossible in this species due to non-resupination, which places the stigma above the anther (gynostemium faces downward) [3,5]. Since *Gymnadenia winkeliana* produces nectar and rewards its insect pollinators/visitors, the pollination method employed is a generalized food-foraging strategy (insects are attracted by food, i.e., nectar) [80,81]. This strategy is largely employed by most of the rewarding orchids, accounting for approximately two-thirds of all species in the family [87,88,89,90,91].

In this strategy, generalist insects are drawn to the sweet vanilla scent emitted by orchids and exploit available food or nutrient sources, such as nectar or floral exudates. Simultaneously, rewarding orchids increase their chances of pollination and seed production due to the insects’ constant food-foraging behaviour, always in search of food sources [92,93,94]. This pollination strategy contributes to the ecological success of both generalist insects and rewarding orchids [95,96,97,98].

*Gymnadenia winkeliana* is found at very high altitudes, ca. 1.900–2.010 m a.s.l., in Bucegi Natural Park, thriving in an alpine habitat characterized by a harsh microclimate. Summers in this region are typically cool, marked by frequent rainfall and storms, with sporadic overnight snowfall being common, particularly during May and June when *Nigritella* Rich. orchids are in full bloom (Figure 2B,C). Consequently, the pollinator communities in this area are rather modest, consisting of fewer species with even fewer insect representatives serving as potential orchid pollinators or visitors. During our pollinator monitoring studies, we encountered several species, as documented in Table 2, including various Lepidopteran, Coleopteran, Hymenoptera and Dipteran insect species. Similar observations were previously reported by Fægri and Van Der Pijl (1979) [99], Vöth (2000) [100] and Claessens and Kleynen (2011, 2016) [3,5]. These data strongly support our initial hypothesis that *Gymnadenia winkeliana* may be a facultative allogamous species.

##### Insect Pollinators and Visitors Encountered

When comparing the insect species from the four major orders, significant differences were observed in the efficiency of pollination of *Gymnadenia winkeliana*. Various ecological aspects of insect pollinators/visitors and pollination systems employed are described below.

**Order Coleoptera Linnaeus, 1758 (beetles)**: Several species of Coleopterans were observed visiting the flowers of *Gymnadenia winkeliana*. These were mostly members of Cantharidae Imhoff, 1856 (soldier beetles) (Figure 8D,E), Chrysomelidae Latreille, 1802 (leaf beetles) and Elateridae Leach, 1815 (click beetles) (Figure 8G) families, primarily phytophagous or pollen-foraging species. Phytophagous beetles are herbivorous insects that feed on plant material, including leaves, stems, flowers and roots. In some instances, they may directly harm orchids by consuming their tissues, leading to reduced plant fitness or even death. While not all phytophagous beetles feed destructively, some species may inadvertently serve as pollinators for orchids. These beetles may visit orchid flowers in search of food or shelter and, in the process, transfer pollen from one flower to another, facilitating pollination. During our studies from 2017 to 2023, there were no instances where Coleopterans were observed carrying pollinia, although they may serve as accidental pollinators for high-altitude orchid species. However, we did observe several pollination events in which *Dactylorhiza viridis* (L.) R.M.Bateman, Pridgeon & M.W.Chase, occurring alongside *Gymnadenia winkeliana*, was efficiently pollinated by *Rhagonycha lignosa* (Müller, O.F., 1764), a member of the Cantharidae Imhoff, 1856 family of beetles frequently found in the same location [1,22,30] (Figure 8B–E,G).

**Order Diptera Linnaeus, 1758 (flies, gnats, mosquitoes)**: Several Dipterans were observed visiting *Gymnadenia winkeliana.* Small Dipterans, such as Empididae Latreille, 1804, (Figure 8A and Figure 9B) are frequent visitors to orchids and efficient pollinators, especially in environments where other pollinators are scarce, such as alpine grasslands and wetlands [28,101] or mountain habitats with cold climates [102,103]. Some species are also predators or carnivores, preying on larvae, but they may also forage on nectar and floral exudates [104,105]. On several occasions, species, such as *Empis ciliata* Fabricius, 1787 (black dance fly) or *Empis trigramma* Wiedemann in Meigen, 1822 (yellow dance fly) from the family Empididae Latreille, 1804 (dagger flies) (Figure 8A and Figure 9B) were observed carrying multiple pollinaria attached to their long proboscides. While dagger flies may not be as specialized as other pollinators, like moths or butterflies, their visits to orchid flowers can still lead to pollination, including both cross-pollination and geitonogamy [27,28,106].

Common flies belonging to the families Anthomyiidae Robineau-Desvoidy 1830 (houseflies), Muscidae Latreille, 1802 (house flies or stable flies) and Syrphidae Latreille, 1802 (hoverflies or syrphids) were frequently observed visiting *Gymnadenia winkeliana* inflorescences, being highly abundant in its specific alpine habitat (Figure 9A–H,J,K). These insects are generalized food foragers and are attracted to any floral attractants in their constant search for food. In several instances, they may inadvertently pollinate orchids as accidental pollinators. However, no species of flies were observed carrying any *Gymnadenia winkeliana* pollinia. Members of the Muscidae Latreille, 1802 family may be considered accidental/potential pollinators of *Gymnadenia winkeliana*, as they were observed previously [3,5,106]. We documented syrphids carrying pollinia of *Dactylorhiza maculata* (L.) Soó, 1962, *Dactylorhiza fuchsii* (Druce) Soó and *Anacamptis coriophora* (L.) R.M.Bateman, Pridgeon & M.W.Chase on multiple occasions (unpublished results) (Figure 8A, Figure 9A–H,J,K and Figure 10H),

**Order Hymenoptera Linnaeus, 1758 (wasps, bees, ants)**: Generally, bees and bumblebees from the family Apidae Latreille, 1802 are considered the most efficient orchid pollinators [1]. However, our observations have shown that they are relatively scarce in the high-altitude habitats specific to *Gymnadenia winkeliana*. This scarcity may contribute to the low frequency of orchid pollination in these areas. During our study, only two instances were recorded in which a single pollinarium was observed attached to the proboscides of *Bombus pratorum* (Linnaeus, 1761) (early bumblebee) and *Apis mellifera* Linnaeus, 1758 (European honey bee) (Figure 9I). Therefore, bees and bumblebees appear to play a minor role in the pollination of *Gymnadenia winkeliana*. While ants from the family Formicidae Latreille, 1809 are not typically regarded as primary pollinators, like moths and butterflies, they may have a minor role in the pollination of certain orchid species, particularly in temperate regions (Figure 8A,F). This is because the pollinaria of temperate orchids are small enough to be removed and transported to another flower by such small insects. We observed several *Gymnadenia winkeliana* inflorescences inhabited by black aphids, members of the superfamily Aphidoidea Geoffroy, 1762. These small sap-sucking insects infest young orchid inflorescences and typically feed on the plant’s sap by piercing the tissues with their needle-like mouthparts (Figure 8F). Ants and aphids often engage in a mutualistic relationship known as trophobiosis [107,108]. Aphids secrete a sugary substance called honeydew, which ants harvest as a food source [109]. In this relationship, ants protect aphids from predators and parasites [108]. As a result, *Gymnadenia winkeliana* inflorescences were frequently visited by ants from the family Formicidae Latreille, 1809, such as *Formica fusca* Linnaeus, 1758 (silky ant), *Myrmica rubra* (Linnaeus, 1758) (common red ant, European fire ant) and *Myrmica schencki* Viereck, 1903 (flower ant). On the orchid inflorescences, ants were observed to gently tap the black aphids with their antennae to stimulate honeydew secretion, while also patrolling the opened flowers foraging for nectar or floral exudates. No ants were observed to directly pollinate *Gymnadenia winkeliana*, but accidental pollination may occur. Myrmecophily (ant pollination), was observed in the case of *Epipactis palustris* (L.) Crantz and *Neottia ovata* (L.) Bluff & Fingerh., which were recorded as being pollinated by ants attending aphid “farms” on their inflorescences [1,106]. *Gymnadenia winkeliana* inflorescences bearing black aphids were also observed to be briefly visited by beetles of the family Coccinellidae Latreille, 1807 (Ladybugs, Ladybirds), such as *Coccinella septempunctata* Linnaeus, 1758 (seven-spot ladybird), and *Hippodamia variegata* Goeze, 1777 (variegated ladybug). Ladybirds are carnivorous insects and typically visit orchids in search of aphid colonies, on which they prey. Although they are frequently encountered on orchid inflorescences, they are not true orchid pollinators but may be considered potential accidental pollinators (Figure 8A,F and Figure 9I).

**Order Lepidoptera Linnaeus, 1758 (butterflies, moths)**: Moths and butterflies have proven to be the most effective pollinators of the high-altitude orchid *Gymnadenia winkeliana*, as well as its sympatric relative *Nigritella nigra* subsp. *bucegiana* Hedrén, Anghel. & R.Lorenz. Similar to all the representatives of subgenus *Nigritella* Rich., both species have a short, rounded spur, making them rather atypical lepidopteran flowers. Nonetheless, they are predominantly pollinated by diurnal butterflies and moths belonging to various families (refer to Table 2). The protruding rostellum and viscidia effectively reduce the spur entrance, resulting in a very small opening for entry [3]. Consequently, only insects with long, slender proboscis, such as moths and butterflies [17,110], can access the nectar secreted in the spur. The strong vanilla scent, striking colouration and significant nectar production serve as efficient attractants for Lepidopterans. We documented ca. eleven/twelve lepidopteran species belonging to seven families, Crambidae Latreille, 1810 (snout moths, grass moths), Erebidae (Leach, 1815) (macromoths), Zygaenidae Latreille, 1809 (burnet or forester moths), Nymphalidae Rafinesque, 1815 (brush-footed butterflies), Adelidae Bruand, 1851 (fairy longhorn moths) and Hepialidae Stephens, 1829 (ghost moths), as efficient pollinators for *Gymnadenia winkeliana* (Figure 10A–G,I–K).

Out of these families, the representants of the family Nymphalidae Rafinesque, 1815 [*Boloria pales* (Denis & Schiffermüller, 1775), (shepherd’s fritillary) (Figure 10A–C,F,K), *Erebia epiphron* (Knoch, 1783) (small mountain ringlet), *Erebia medusa* (Denis & Schiffermüller, 1775) (woodland ringlet)] were the most successful, followed by Zygaenidae Latreille, 1809 [*Zygaena exulans* (Reiner & Hohenwarth, 1792) (Scotch burnet) (Figure 10D), *Zygaena loti* (Denis & Schiffermüller, 1775) (slender Scotch burnet) (Figure 10J)], Hepialidae Stephens, 1829 [*Pharmacis carna* (Denis & Schiffermüller, 1775) (ghost moths, furry moth) (Figure 10E,G)] and Crambidae Latreille, 1810 (*Crambus* sp. Fabricius, 1798 (snout moths), *Crambus uliginosellus* Zeller, 1850 (sod webworms)]. Among all lepidopteran species, *Boloria pales* (Denis & Schiffermüller, 1775) proved to be the most prevalent and active pollinator present within the specific habitat of *Gymnadenia winkeliana* (Figure 10A–C,F,K). The shepherd’s fritillary butterflies were documented visiting the inflorescences carrying multiple yellow pollinaria attached to the long proboscides (Figure 10A,B,F). Butterflies and moths pollinators stay for extended periods on the same inflorescence (up to 3–5 min), systematically moving from one flower to another, causing a high degree of geitonogamy (personal observation). Our findings are consistent with observations/reports by Ziegenspeck (1931) [111] and Muller (1874) [112] who classified species of the polyploids of the *Gymnadenia nigra*-group as lepidopteran species. The majority of the observed butterflies were diurnal species. Additionally, previous studies conducted by Schiestl & Schlüter (2009) [113] also documented that orchids of the former genus *Nigritella* Rich. are pollinated by Lepidopterans with medium-sized to short proboscises. From our observations, we conclude that this putative facultative allogamous species primarily relies on diurnal butterflies and moths for cross-pollination, particularly those belonging to the families Nymphalidae Rafinesque, 1815, Zygaenidae Latreille, 1809, Hepialidae Stephens, 1829 and Crambidae Latreille, 1810. It is noteworthy that *Boloria pales* (Nymphalidae Rafinesque, 1815) and moths of the family Crambidae Latreille, 1810 were also noted as efficient pollinators for neighbouring sympatric orchid species, such as *Gymnadenia conopsea* (L.) R.Br., *Pseudorchis albida* (L.) Á.Löve & D.Löve and *Pseudorchis albida* subsp. *tricuspis* (Beck) E.Klein [22,28,30,101].

In conclusion, Lepidopterans were the dominant taxa of pollinators in regards to the overall number of species recorded, proving to have stronger interactions with *Gymnadenia winkeliana*, potentially suggesting a more specific relationship between the two parties. The newly identified species, *Gymnadenia winkeliana*, demonstrates a preference for several species of moths and butterflies as pollinators, with *Boloria pales* (Nymphalidae Rafinesque, 1815) being the most frequent and efficient visitor of the inflorescences (Figure 10A–C,F,K). Despite possessing atypical lepidopteran flowers characterized by a short, rounded spur, butterflies and moths proved to be the primary (potential) pollinators of the alpine *Gymnadenia winkeliana*. Our observations of the pollinator community within the restricted study area support our initial hypothesis that *Gymnadenia winkeliana* may be regarded as a putative generalist pollinator, capable of employing facultative allogamy when pollinators are available. Generalized pollination strategies have been associated with the extensive diversification of orchid species (Ray and Gillett-Kaufman, 2022). These observations are in line with previous studies that show that species of the former genus *Nigritella* Rich. may potentially switch from asexual (apomixis) to sexual reproduction (allogamy). Hence, their potential for employing both reproduction strategies provides them with greater reproductive flexibility, enabling them to adapt to and colonise diverse new habitats and overcome various reproductive challenges.

### 3.4. Potential Occurrence of Intra- and Intergeneric Hybrids

Despite the diverse pollinator community in the study area and frequent interactions between various insect species and sympatric orchids, no hybrids were observed between *Gymnadenia winkeliana* and other diploid (2n = 2x = 40) related species, like *Pseudorchis albida* (L.) Á.Löve & D.Löve, *Pseudorchis albida* subsp. *tricuspis* (Beck) E.Klein and *Gymnadenia conopsea* (L.) R.Br., the only representative of the genus *Gymnadenia* R.Br. in the habitat. While hybrids could potentially be produced, as pollinators were observed carrying *Gymnadenia winkeliana* pollinaria between sympatric individuals, there appears to be a post-pollination mechanism (either pre- or postzygotic) acting as a barrier, thus preventing cross-pollination and hybrid formation. One possible explanation could be the notable disparity in population sizes between the parental species. While *Gymnadenia winkeliana* was found in relatively large numbers, the population of *Gymnadenia conopsea* (L.) R.Br. consisted of only 2–4 mature or flowering individuals at the specific location. Additionally, although *Pseudorchis albida* subsp. *tricuspis* (Beck) E.Klein was more abundant in the habitat, there were considerable distances between its populations and those of *Gymnadenia winkeliana*, spanning approximately 30–50(60) meters. This spatial separation could act as a barrier to cross-pollination, especially in a habitat characterized by a specific microclimate, characterized by frequent rains, strong winds and low temperatures, which may limit the movement of pollinators over longer distances.

Consequently, it is important to note that the processes involved in the formation of such hybrids are still under investigation, and the possibility of their occurrence remains open.

**Figure 11:**
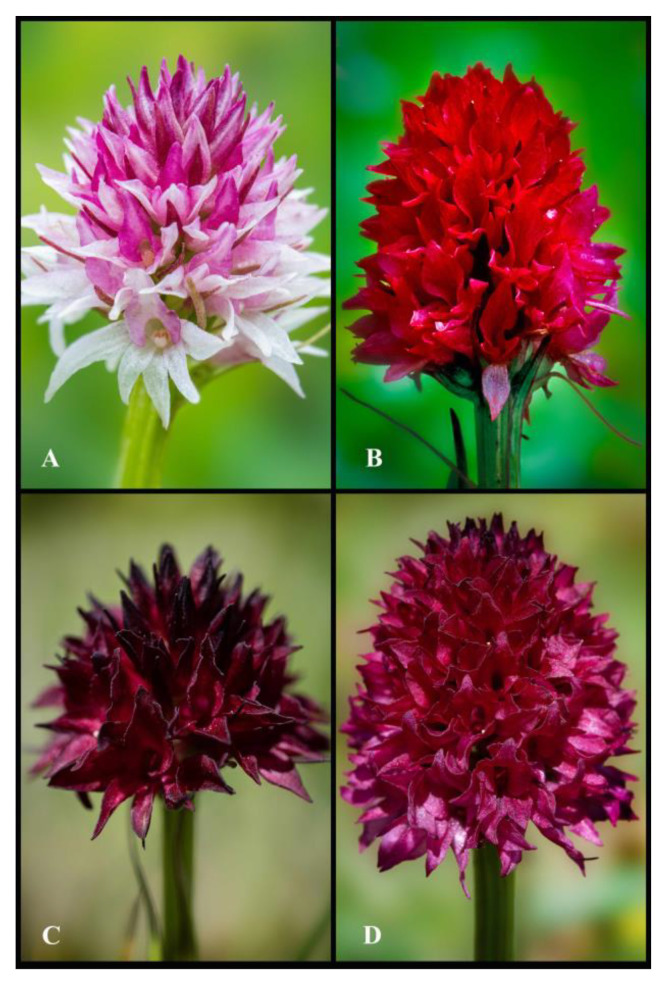
Members of the former genus *Nigritella* Rich. present in Romania. (**A**) *Gymnadenia carpatica* (Zapał.) Teppner, E.Klein & Zag. (**B**) *Gymnadenia miniata* (Crantz) Janch. (**C**) *Gymnadenia austriaca* (Teppner & E.Klein) P.Delforge. (**D**) *Nigritella nigra* subsp. *bucegiana* Hedrén, Anghel. & R.Lorenz. Illustration and photos by Nora E. Anghelescu, June–July 2017–2023 BNP, Romania.

Other highly morphologically distinctive members of the subgenus *Nigritella* Rich. present in Romanian Carpathians are the diploid (2n = 2x = 40) *Gymnadenia carpatica* (Zapał.) Teppner, E.Klein & Zag., a Romanian sub-endemic present in Eastern Carpathians, exclusively confined to Northern Romania and Ukraine (Figure 11A), and the tetraploids (2n = 4x = 80) *Gymnadenia miniata* (Crantz) Janch, a uniformly bright-red species (Figure 11B) and the dark-red to dark-brown *Gymnadenia austriaca* (Teppner & E.Klein) P.Delforge (Figure 11C) and *Nigritella nigra* subsp. *bucegiana* Hedrén, Anghel. & R.Lorenz (Figure 11D), all present in Southern and Eastern Carpathians. The study of the former genus *Nigritella* Rich. continues as ongoing work, with the list remaining open to potential new taxonomical additions, still waiting to be discovered within the vastness of the Romanian Carpathians, a region that remains relatively unexplored to date.

## 4. Materials and Methods

### 4.1. Sites Studied

The studies were conducted within the alpine areas of the Bucegi Natural Park ROSCI0013, a protected area IUCN category V (Protected Landscape, Law No. 5, 6.03.2000), part of the Natura 2000 site, covering Prahova, Dâmbovița and Brasov Counties, Southern Carpathians, Central Romania. The Park has an area of ca. 32.663 ha/326.63 km^2^, with the highest elevation (elev.) at Omu Peak of 2.505–2.514 m a.s.l (above sea level) (see previous orchidological studies within the same protected area by [8,101] (Figure 1A–C and Figure 2B,C).

### 4.2. Populations Studied

The *Gymnadenia winkeliana* population was first discovered at locus classicus, in 2005, during an orchidological expedition, containing approximately 50–55 individuals (information obtained from Dan Anghelescu, personal communication, 2005–2016). Over time, the population size increased consistently, reaching approximately 80–100 individuals between 2012 and 2020, and approximately 120–130(140) individuals from 2020 to 2023. The initial population numbers may have been higher, considering that the areas were constantly utilized as cattle fields and a portion of the vegetation had already been destroyed by the grazing animals (Figure 1C, red dots and Figure 2A).

### 4.3. Extent of Occurrence (EOO)

The population was found to be spread over an alpine plateau (grassland) with an EOO of ca. 2.5–5 km^2^, locus classicus GPS of 45°23′06.82″ N, 25°28′27.81″ E, elev. 2.000–2.010 m a.s.l., currently in Dâmbovița County, Bucegi Mountains Natural Park ROSCI001, Southern Carpathians, Romania (Figure 1C, red dots).

### 4.4. Study Time Frames

June–July 2005–2023.

### 4.5. Species Studied

*Gymnadenia winkeliana* individuals were studied according to morphology, habitat, flowering time, geographic location and accessibility. The plants were collected between 29 June and 5 July 2023 under the permit granted by the Bucegi Natural Park Administration: Research Permit No. 1887/CAN/22.07.2021–2023; APN–Bucegi (RO: Administratia Parcului Natural Bucegi) (Figure 2B,C, Figure 3A–F and Figure 4A–L).

### 4.6. Morphological Measurements

Measurements of the vegetative and floral parts were made from living plants and fresh flowers. To describe this newly found population as comprehensively as possible, 175 morphological features were directly studied and measured from living plants and flowers. The morphological characteristics used for the study included most of the characteristics used previously [8,28]. Special attention was focused on the characteristics that proved to have taxonomic significance, particularly those involving distinctive details in the morphology of the leaves, perianth, labellum and gynostemium. Only flowering parts of the plants were sampled, leaving the vegetative parts for persistence and continued growth. The 223 characteristics scored encompassed the morphology of every organ of the plant, cytology and breeding system. No organ was represented by fewer than three characteristics. The characteristics (listed in Table 1) described in detail are the roots (8), stem (9), leaves and bracts (36), inflorescence and flower (16), sepals and petals (17), labellum and spur (30), gynostemium (13), anther (13), pollinia and pollinarium (27), stigma (19), ovary (12), fruit (6), seed and embryo (15), chromosomes (2), flowering time and reproductive strategies (7).

### 4.7. Pollination Monitoring

Monitoring was conducted for a total of 5–6 h per day, mostly between June–July (2017–2023), when most of the flowers were in full anthesis. The observer (NA) initially positioned themselves approximately 2–3 meters away from the subjects, whether they were in groups or individual plants. Digital photographs and insect pollinators/visitors records were taken upon observing different insects patrolling or approaching the flowers. It is important to note that no insects were collected or harmed in any way during the study. A comprehensive list of insect pollinators and visitors is given in Table 2.

### 4.8. Explant Collection

Wherever possible, several bract-ovary-flower units were removed from a position approximately one-third to halfway from the base of the inflorescence (Figure 5C–E,I,J). This was performed to minimize the impact of the pink colour gradient and the decrease in flower size observed from the base to the apex of the inflorescence, a characteristic of this species. The removal of fewer flowers/inflorescences does not affect the viability of the plants, since the leaves and roots remain intact. The plants collected in the field were kept in vials with fresh water, at 4 °C, for approximately 14 h, before being submitted to stereomicroscopy for biometrical measurements. Immature seeds (from indehiscent fruit/seed capsules) were collected 5–6 weeks after the peak of antheses. Seed capsules were opened and fresh seeds were submitted to stereomicroscopy for biometrical measurements.

### 4.9. Stereomicroscopy

The flower–bract units were analysed and biometrically measured under a stereomicroscope, before and after dissection (for floral parts and reproductive organs biometry). The stereomicroscope(s) had a transmitted light 2x–8x Trinocular Boom Stand Stereo Zoom Microscope + 9 MP Camera. Camera information was as follows: Calibration = 0.005 millimetres/pixel; capture format = 2048 × 1536, Full Frame HQ; Gamma = 1.06; Gain = 1.0×; Exposure = 44.5 ms; Auto exposure = Off; Image typ = Colour; Shading = (None); Sharpening = Medium; Black clip = 6; White clip= 253. Microscope information was as follows: Leica M125 C, 12.5:1 zoom; 8× Main Objective Magnification = 1 to 8.0×; Zoom Magnification = 1.01; Visual Magnification = 10.10; Video Magnification= 0.64. The resulting images were recorded digitally for subsequent manipulation in Adobe Photoshop.

### 4.10. Digital Photographic Equipment

Digital images of individual plants and floral parts were taken using Nikon D3 and Nikon D850 camera bodies equipped with Nikon Micro NIKKOR 60 mm and NIKKOR 24.0–70.0 mm lenses. Additional equipment included a Manfrotto Tripod and Litra Torches 2.0s. An adapted Helion FB tube was used for automated focus bracketing. The images were analysed using Adobe Photoshop^®^ CC 2024, Zerene Stacker Software, Version 2021-11-16 [78].

### 4.11. Maps

The map was created using ArcGIS Pro 3.1 software; the maps and elevation services were provided by the entities mentioned in the copyright [8]

## 5. Conclusions

This monophyletic group [9,46,114], which encompasses numerous recently evolved species, is currently undergoing an evolutionary radiation. This radiation is driven by a wide range of factors, including genotypic (such as genetic and epigenetic factors, genetic drift), phenotypic (ecophenotypic) and environmental influences (such as habitat alterations, climate fluctuations and the presence or absence of true pollinators and specific mycorrhizal associations). Hence, the remarkable ability of European vanilla orchids to exhibit diverse phenotypic responses to environmental demands has led to significant and rapid changes in the taxonomy of the genus *Gymnadenia* R.Br. in recent years, driven by the emergence of micro-endemic populations with varying reproductive strategies. Constantly evolving morphological adaptations to novel and isolated habitats are frequently documented, often sparking extensive discussion and making the newly emerged taxa the focus of attention. Consequently, in the last decade, the aggregate *Gymnadenia* R.Br. has become one of the most taxonomically complex and debated orchid genera in Europe.

## 6. Taxonomic Treatment

Our morphometric data and detailed morphological characterization demonstrate that *Gymnadenia winkeliana* is a highly distinct taxon, significantly differentiating from all other *Gymnadenia* R.Br., species part of the Romanian flora. Altogether, we find it appropriate to recognize the Southern Carpathian population of *Gymnadenia winkeliana* as a separate species. Its formal description follows below.

***Gymnadenia winkeliana* N.Anghelescu, L.Balogh, M.Balogh & N.Kigyossy, 2024 sp. nov.** (Figure 2, Figure 3, Figure 4, Figure 5, Figure 6, Figure 7, Figure 8, Figure 9 and Figure 10).

**Diagnosis**: The endemic *Gymnadenia winkeliana* differs from all other Romanian *Gymnadenia* R.Br. species by its smaller habitus size, hemispherical, roundish two-coloured pinkish, pale violet (bluish hued) inflorescence, white-to-pale-pink small flowers, short flowering period (up to 4–5 maximum 8 days) and exclusively alpine/high altitude area of occurrence (2.000–2.010 m a.s.l.).

**Holotype**: România, Southern Carpathians, Bucegi Natural Park ROSCI001 Natura 2000 (Dâmbovița, Moroeni, Bucegi); alpine grassland, calcareous conglomerate, leg. Nora E. Anghelescu sub No. 1–4 ex. specimen typorum: GPS: 45°23′06.82″ N, 25°28′27.81″ E, elev. 2.000–2.004 m a.s.l.; period: 29.06.2023–5.07.2023, fl. 25.06–10.07; voucher specimens were deposited at the Herbarium of the University of Agriculture and Veterinary Medicine, Bucharest, NE Anghelescu, USAMVB Herbarium barcode: 40102 (Holotype: USAMV). Additionally, the holotype was confined to ca. 900–1000 digital images © 2017–2023 NEA, LB, NK, deposited in private image databases.

**Icon. hoc loco**: Figure 5, Figure 6 and Figure 7 (Holotypus), Figure 2 (Holotypus, paratypi), Figure 3, Figure 4, Figure 8, Figure 9 and Figure 10.

**Icon. altera**: De Angelli & Anghelescu (2020): 104–105, 5 Figs.; sub ‘N. cf. widderi’ [1]; De Angelli (2022): 241, Figure 1 sub ‘N. cf. widderi’ [115]; De Angelli (2021): Front cover OD (Orchid Digest), Vol. 85–3 sub ‘N. cf. widderi’ [116].

**Etymology**: The specific epithet ‘*winkeliana*’ was given in memory and honour of Dutch botanist Gab van Winkel (May 1955–September 2023), Editor-in-chief of Orchideeën, the official magazine of the Nederlandse Orchideeën Vereniging (Dutch Orchid Association) and director of the prestigious official website of the European Orchid Council (EOC). Throughout his life, he devoted himself primarily to the study and documentation of orchids. On several occasions, he travelled to Romania where he researched several orchid species, including the genus *Gymnadenia* R.Br. Concurrently, he played a pivotal role in supporting and advocating for orchid-related endeavours, such as books, exhibitions, articles and narratives, all while fostering a large, international community of orchid enthusiasts—‘*A great man with a great heart*’ (Manuel Lucas, 2023 [117]).

**Description**: Described exclusively from living plants and flowers.

*Gymnadenia winkeliana* is a terrestrial, perennial, rhizomatous, autotrophic, sympodial herbaceous geophyte, 85–105(140) mm tall, including the inflorescence. ***Rhizome*** (hypogeal part of the stem) 5–7(8) × 3–4(6) mm (length × diam.), short, thick, very compressed. It produces 2–6(8), 10–45(55) × 1.1–1.8 mm, cylindrical, thick, elongate ***adventitious roots*** and 2(3), 6.7–9.7(10) × 3.8–5.3(6.2) mm flattened, deeply digitate (divided for at least half of their length) ***root-tubers*** (usually formed by the tuberization of short adventitious roots). ***Flowering stem*** (epigeal part of the stem), unique, 85–105(140) × 2.8–3.1(4) mm, slender, erect, spindly, flexuous, ridged, solid, vivid-green, non-pigmented (anthocyanins absent), glabrous (trichomes or glandular hairs absent). ***Basal leaves*** 5–10(12), 33–65(82) × 26–48(53) mm (length × width), narrowly lanceolate, moderately to strongly keeled, erect to spreading, to a subtended angle of c. 40°–45° relative to the stem, grass-like, vivid-green, presenting a smooth, non-pigmented (anthocyanins absent), non-marked (purple maculae absent) surface, with a faint median vein, faint parallel venation (multiply ribbed) and entire, straight (not undulating) margins, acute/tapering at the tip, forming a summer-green basal rosette (it emerges above ground exclusively during the vegetative season). ***Cauline leaves*** 4–7(8), 32–56(69) × 21–35(41) mm, alternate/distichous, uniformly distributed along the stem, triangular-lanceolate, sheathing, vivid-green with red (purple)-brown margins and tips (anthocyanins present), with a non-marked (purple maculae absent) textured surface with median venation (midrib), partially arched and relatively stiff. Upmost cauline leaf shorter, longer than the basal flower. Cauline leaves hypoamphistomatic, edged with fine, hyaline/translucent, conical, closely-spaced, irregular tooth-like ***papillae***. ***Flower bracts*** 6.3–8.3(12.8) × 2.3–3.4(3.9) mm, narrowly triangular, erect to angled, longer than the flowers, green, strongly purple-brownish pigmented at the tip and margins (anthocyanins present), textured, acute, triangular elongate to narrow lanceolate, with numerous ***anomocytic stomata*** on the adaxial side and with margins moderately papillate (closely-spaced) to finely serrated. ***Bract hyaline papillae*** 0.04–0.14(0.28) mm highly variable in size, forming an irregular/serrated margin. Basal bracts horizontally spreading to a subtended angle of c. 90° relative to the stem (perpendicular to the stem). ***Inflorescence*** 10.5–17.5(20.5) × 8.5–10.2(12.5) mm, dense, floriferous, terminal raceme, pyramidal at the beginning of anthesis, spherical to hemispherical in full anthesis, acropetal (opening from the base upwards), two-coloured, displaying a gradient of pink hues—white/pale pink (basal flowers) or pale-pink/dark-pink (top flowers and buds). ***Flowers*** 40–60(80), 5.2–6.8(7.5) × 4.1–5.8(6.1) mm (length × width), star-like, roundish, white/pale pink (basal flowers) or pale-pink/dark-pink (top flowers and buds), sessile, chasmogamous, wide opened, non-resupinate (labellum directed upwards). ***Scent*** present, sweet, vanilla-like, intense during morning hours (pers. obs.). ***Lateral sepals*** 2, 5.6–6.9(7.1) × 0.8–1.3(1.9) mm, median sepal 5.9–7.0(7.4) × 1.1–1.6(2.1) mm, petaloid (petal-like), approximately equal in size, white/pale pink (basal flowers) or pale-pink/dark-pink (top flowers and buds), elliptic to elongate-lanceolate, arched, strongly acuminate/acute, slightly concave, with smooth surface. Lateral sepals spreading horizontally to slightly downward tipped, ***median sepal*** pointing (vertically) downward. ***Lateral petals*** 2, 4.9–5.4(6.1) × 0.4–0.7(1.1) mm, sepaloid (sepal-like), equal in size, white/pale pink (basal flowers) or pale-pink/dark-pink (top flowers and buds), elongate-lanceolate, arched/flared, strongly acuminate at the tip, narrower than the sepals, spreading, pointing downward to a subtended angle of c. 40°–45° relative to the flower axis. ***Labellum*** 6.1–6.5(7.1) × 2.2–2.8(3.1) mm, (length × width), white (basal flowers) to pale-pink or dark-pink topmost flowers (buds), non-resupinated, upwards oriented, acuminate, rhomboidal, more-or-less planar to slightly convex, margins slightly undulate, lateral constriction pronounced, median ridge/vein absent, markings/spots absent, shallowly 3-lobed, with a bulbous base 1.5–1.7(1.9) mm. Median lobe triangular, elongate. Lateral lobes scalloped, roundish, bent upwards. Apical part (epichile) 2.3–2.5(2.8) × 2.2–2.8(3.1) mm, heart-shaped, flared, erect/arched upwards (due to non-resupination). Middle part (mesochile) (0.1/0.2)0.6–0.7(0.78) mm wide, tube-like/saddle-like junction formed by the narrowing edges of lateral lobes, which almost touch each other. Basal part (hypochile) 1.5–1.7(1.9) wide, bulbous, roundish, containing the gynostemium (reproductive organ). ***Spur*** 2.5–2.9(3.1) × 1.9–2.5(2.9) mm (length × diam.), short, broad, saccate-shaped, spherical-ovoidal, whitish, translucent-white, straight (not curved/arched). ***Nectar*** present in moderate amounts, filling up to ¼–½ spur length (nectar-rewarding species). ***Gynostemium*** 5.2–6.1(6.3) × 2.9–3.1(3.4) mm, thick, cylindrical, translucent to yellowish, faintly purple pigmentated (slight traces of anthocyanins present), horizontally to downwards oriented inside the flower (due to non-resupination). ***Staminodes*** absent. ***Auricles*** 2, 0.5–0.7(1.1) × 0.3–0.5(0.8) mm, placed laterally of the gynostemium (gynostemial auricles), prominent, ovoidal to spherical, verrucose, translucent white, non-pigmented (anthocyanins absent). ***Clinandrium*** absent. ***Anther*** 1, 2.9–3.2(3.7) × 1.2–1.5(1.8) mm, fertile, bithecal (contains two parallel, identical thecae, termed as chambers/loculi), elongate, translucent white to yellowish. ***Thecae*** 2, 3.2–3.8(4.2) × 1.3–1.6(1.8) mm, parallel, elongate, translucent-white to yellowish, non-pigmented (anthocyanins absent), each containing 1 massulate ***pollinarium***. ***Connective tissue*** present, connecting the two thecae, roundish, translucent-white to yellowish, with a purple-pigmented roof (anthocyanins present). ***Anther cap*** absent. ***Pollinia*** 2, 3.1–3.5(4.1) × 1.3–1.6(1.8) mm, massulate, moderately compact, one in each theca, ovoidal-elongate, yellow, with no stigmatic contact (prevented by the strongly developed rostellum and median rostellar fold/median rostellar lobe). ***Massulae*** 48–60(78), 0.5–0.53(0.6) × 0.45–0.48(0.5) mm (length × width of each massula), formed of compact blocks of pollen grains (tetrads) held together by elastoviscin, ovoidal, roundish, yellow. ***Caudicles*** present, 1.2–1.6(1.4) × 0.1–0.12 (length × diam.), long (>30% of length of pollinium), yellow to translucent-yellow, connecting the pollinia to the viscidium/viscidial disk. ***Viscidium***/viscidial disk present, 0.7–0.83(1.1) mm diam., enclosed in a rudimentary bursicle (young flowers); naked/free (not enclosed in a membranous bursicle) in older flowers, round-ellipsoidal/approximately circular, translucent white, covered in viscous viscidial exudate that adheres to insects’ mouthparts/proboscides. ***Pollinaria*** 2, placed proximal and parallel on gynostemium, each pollinarium formed of one massulate pollinium, caudicle and viscidial disk. There are two pollinaria/anthers, one in each theca, containing all the pollen of the flower. ***Stigma*** 1.9–2.3(2.8) × 4.8–5.1(5.4) mm (height × width), situated below the anther, concave, elliptic, translucent white, wet (covered in stigmatic exudate), perpendicular to the axis of the gynostemium, three-lobed: one median (rostellum) and two lateral lobes. ***Lateral lobes*** 2, 2.4–2.6(2.9) × 1.2–1.4(1.6) mm (length × width), lappets-like/flaps-like spreading laterally, flanking the stigmatic cavity, fertile, concave, with prominent lower rim. ***Rostellum*** (median lobe), present above spur entrance, roof-like, not fertile, translucent white, with a prominent median ***rostellar fold*** 2.3–2.4(2.6) mm in height, separating the anther from the stigmatic cavity, thus preventing autogamy/self-pollination. Stigmatic surface and lateral stigmatic lobes covered in abundant viscous, stigmatic exudate. ***Bursicles*** membranous, rudimentary/reduced. ***Ovary*** 3.1–3.3(3.6) × 1.6–1.8(1.9) mm (length × width), unilocular, epigynous (enclosed in the receptacle, with the floral parts arising above it), ovoidal, green, non-resupinate (untwisted), sessile, glabrous, subtended to an angle of c. 45° relative to the stem. ***Flower pedicel*** absent (sessile flowers). ***Placentation*** parietal. ***Ovule*** 0.42–0.68(0.74) × 0.28–0.32(0.39) mm, anatropous, tenuinucellate, ovoidal elongate, translucent-white to whitish-green.

**Flowering time**: Synchronized, from late June to mid-July.

**Flowers longevity**: Spans over 5–8 days.

**Fruit**: 3.5–3.8(3.9) × 1.8–1.9(2.1) mm (length × width), elongate-ovoidal green pod, subtended to an angle of c.45° relative to the stem, with three highly pronounced longitudinal ridges (three lines of dehiscence).

**Fruiting**: June–July.

**Fruit set**: 40–62%.

**Seeds maturation**: July–September.

**Seed capsule**: 3.5–3.9(4.1) × 1.8–1.9(2.2) mm, brownish, ovoidal-elongate, slightly larger than the fruit pod, presenting three longitudinal ridges of dehiscence.

**Capsule dehiscence**: August–September.

**Seeds**: 2.1–2.3(2.6) × 1.02–1.06(1.1) mm, minute, numerous (microspermy), elongate-ovoidal to fusiform, tapering from the middle to the tips, covered in a brownish testa. Testa external ornamentation reticulated.

**Embryo**: 0.63–0.75(1.9) × 0.46–0.61(0.86) mm, whitish-yellow, ovoidal, roundish, lacking endosperm, placed centrally within the seed testa.

**Cytogenetics**: 2n = 4x = 80 (putative tetraploid)–chromosome no. is still under investigation.

**Reproductive biology**: Facultative allogamy (sexual) and facultative apomixis (asexual); still under investigation.

**Habitat**: *Gymnadenia winkeliana* prefers alpine grassland on calcareous/alkaline nutrient-poor (oligotrophic to mesotrophic) substrates, occurring sympatrically with various alpine species, such as *Alchemilla flabellata* A.Kern., *Antennaria dioica* (L.) Gaertn., *Arabis alpina* L., *Biscutella laevigata* L., *Carex sempervirens* Vill., *Cerastium arvense* L., *Cerastium holosteoides* Fr., *Clinopodium alpinum* (L.) Kuntze, *Dianthus glacialis* Haenke, *Erigeron neglectus* A.Kern., *Festuca* Tourn. ex L. spp., *Gentiana verna* L., *Leontodon hispidus* L., *Pedicularis verticillata* L., *Pilosella aurantiaca* (L.) F.W.Schultz & Sch.Bip., *Pinguicula vulgaris* L., *Sesleria* Scop. spp., *Thymus pulegioides* L. and many others [23]. At the same time, *Gymnadenia winkeliana* occurs in sympatry with other alpine orchid species, such as *Dactylorhiza viridis* (L.) R.M.Bateman, Pridgeon & M.W.Chase, *Chamorchis alpina* (L.) Rich., *Gymnadenia conopsea* (L.) R.Br., *Pseudorchis albida* (L.) Á.Löve & D.Löve [22] and *Pseudorchis albida* (L.) Á.Löve & D.Löve subsp. *tricuspis* (Beck) E.Klein [30].

**Variability**: Morphological features proved highly consistent, with most vegetative and floral characteristics (height, flower colour) showing constancy and minimal variability within the population. In some individuals, the inflorescence shape may shift toward sub-cylindrical (peak of anthesis) and the basal flowers towards deeper shades of pink.

**Locus classicus**: *Gymnadenia winkeliana* is endemic to the restricted original (holotype) geographic area located in central Bucegi Mountains Natural Park ROSCI001, a protected area included within Natura 2000, IUCN category V (Protected Landscape, Law No. 5, 6.03.2000); GPS: 45°23′06.82″ N, 25°28′27.81″ E, elev. 1.990–2.010 m a.s.l.; currently in Dâmbovița County, Southern Carpathians, Central Romania. This species requires further observation to determine whether other known populations are present in other areas within the Bucegi Mountains Natural Park protected area (Figure 1C).

**Population counts**: Approximately 120–130(140) individuals (n < 200), as recorded between 06–07.2005–2023.

**Dormancy periods**: Span over one vegetative season.

**Area of occupancy (AOO)/Extent of Occurrence (EOO)**: Greater distribution area ca. ca. 2.5–5 km^2^; (micro-endemism); GPS: 45°23′06.82″ N, 25°28′27.81″ E, elev. 2.000–2.010 m a.s.l.; currently in Dâmbovița County, Bucegi Mountains Natural Park ROSCI001.

**Examined material**: Romania. Bucegi Mountains Natural Park ROSCI001 protected area Natura 2000: alpine grasslands areas of higher Dâmbovița County, Southern Carpathians, elev. 2.000–2.010 m a.s.l.; 25 June–10 July 2017–2023, fl. ca. 20–29 June 2023.

**Voucher**: Voucher specimen deposited at the Herbarium of the University of Agriculture and Veterinary Medicine, Bucharest, NE Anghelescu, USAMVB–barcode 40102.

**Proposed conservation status**: **Endangered** (**EN**). *Gymnadenia winkeliana* is an endemic currently reported exclusively from the Bucegi Natural Park ROSCI001 protected area Natura 2000, Dâmbovița County, Southern Carpathians, Romania. As of 07.2023, the *Gymnadenia winkeliana* population contains a total of ca. 120(140) individuals, occurring within an area no greater than 8–10.5 km^2^ (micro-endemism). Nevertheless, we take into consideration that more future research in other subalpine and alpine areas of the park may lead to the discovery of new populations of *Gymnadenia winkeliana*. In recent years, Bucegi Natural Park has been found to host several rare orchid species, including *Ophrys apifera* Huds., *Ophrys scolopax* Cav. subsp. *cornuta* (Steven) E.G.Camus, *Ophrys insectifera* L., [118] and *Cypripedium calceolus* L., [119]. Additionally, the park has been the site of discovery for several new-to-science taxa, such as the newly identified *Epipactis bucegensis* N.Anghelescu, L.Balogh and M.Balogh, sp. nov., 2023 [8] and the rare inter-generic orchid hybrid *× Dactylodenia sinaiensis* N.Kigyossy, N.Anghelescu, L.Balogh & Mih.Balogh, nothosp. nov., 2023 (a naturally occurring hybrid between *Dactylorhiza saccifera × Gymnadenia conopsea*) [101]. It is crucial to emphasize that the micro-endemic species *Gymnadenia winkeliana* is confined to an area that is susceptible to rapid destruction due to uncontrolled overgrazing and increased anthropogenic activities, including cattle farming, tourism and recreational resort development. In line with the EU Biodiversity Strategy (2020–2050), which aims to restore natural environments by halting the destruction of ecosystems and loss of biodiversity [120], it is imperative to implement effective measures to protect and conserve these fragile habitats that host rare endemic species [1,22,101,106]. Since this rare orchid occurs exclusively in a localized population of low densities, it clearly needs to be protected if this species is to persist.

Consequently, we propose that *Gymnadenia winkeliana*, which is exclusively confined to one mountain range (Bucegi Mountains), be classified as ‘Endangered’ (EN) according to the Red List criteria established by the IUCN Standards and Petitions Committee [121].

## Figures and Tables

**Table 1:** Morphometric/biometric measurements, pollinator, flowering phenology, chromosomes and reproductive strategy of the newly identified species *Gymnadenia winkeliana*, prev. used [8,27,28].

Vegetative and Floral Organs	Characteristics/Features Measured	Biometric Measurements (Millimetres)
Rhizome		
	Rhizome shape	Short, compressed
	Rhizome length	5–7(8)
	Rhizome diam.	3–4(6)
Adventitious Roots		
	Adv. roots shape	Cylindrical, thick, elongate
	Adv. roots no.	2–6(8)
	Adv. roots length	10–45(55)
	Adv. roots diam.	1.1–1.8
Root-tubers		
	Root-tubers shape	Deeply digitate, flattened
	Root-tubers no.	2(3)
	Root-tubers length	8.7–9.7(10)
	Root-tubers diam.	3.8–5.3(6.2)
Stem		
	Stem architecture	Slender, ridged, solid
	Stem colour	Vivid-green
	Stem height	85–105(140)
	Stem diameter	2.8–3.1(4)
	Stem anthocyanins	Absent
	Trichomes (Glandular hairs)	Absent
Basal Leaves		
	B. leaves distribution	Forms a basal rosette
	B. leaves shape	Narrowly lanceolate, moderately to strongly keeled
	B. leaves colour	Vivid-green
	B. leaves texture	Smooth
	B. leaves angle relative to the stem	Erect to spreading, to a subtended angle of c. 40°–45° relative to the stem
	B. leaves no.	5–10(12)
	B. leaves length	33–65(82)
	B. leaves width	26–48(53)
	B. leaves margins	Entire, straight (not undulating)
	B. leaves venation	Faint median vein and faint multiple ribs (parallel ribs)
	B. leaves purple markings/spots	Absent
	Apical hooding	Moderate to strong, tapering
Cauline Leaves		
	Distribution on stem	Uniform
	Phyllotaxy (viewed vertically)	Alternate/distichous, sheathing the stem
	C. leaves shape	Triangular lanceolate
	C. leaves no.	4–7(8)
	C. leaves length	32–56(69)
	C. leaves width	21–35(41)
	C. leaves margins	Papillate (closely-spaced)
	C. leaves venation	Distinct median vein (midrib)
	C. leaves anthocyanins	Present, red (purple)-brown margins and tips
	C. leaves purple markings/spots	Absent
	Upmost c. leaf	Shorter, bract-like
Bracts		
	Bracts shape	Narrowly triangular
	Bracts colour	Green, strongly purple-brownish pigmented at the tip and margins
	Bracts length	6.3–8.3(12.8)
	Bracts width	2.3–3.4(3.9)
	Bracts margins	Moderately papillate (closely spaced) to finely serrated
	Papillae size	0.04–0.14(0.28)
	Bracts anthocyanins	Strongly purple-brownish pigmented
	Bracts texture	Textured, with numerous stomata on the adaxial side
	Basal bracts	Foliose, greatly exceeding flowers
Stomata		
	Distribution	Hypoamphistomatic (preponderant on the abaxial surface)
	Type	Anomocytic
	Stomatal length	0.21–0.24(0.37)
	Stomatal width	0.18–0.21(0.29)
Inflorescence		
	Infl. type	Terminal raceme
	Infl. density	Dense, floriferous
	Infl. shape	Ovoid, hemispherical
	Infl. colour	Two-coloured—white/pale pink (basal fl.) or pale pink/dark pink (top fl. and buds.)
	Infl. length	10.5–17.5(20.5)
	Infl. diameter	8.5–10.2(12.5)
	No. of flowers	40–60(80)
Individual Flower		
	Flower type	Chasmogamous, wide-opened
	Flowers shape	Star-like, roundish
	Flower resupination	Non-resupinate
	Flower length	5.2–6.8(7.5)
	Flower width	4.1–5.8(6.1)
	Flower colour	Two-coloured—white/pale pink (basal fl.) or pale pink/dark pink (top fl. and buds.)
Scent		
	Present	Moderate
	Fragrance	Sweet, vanilla-like
Sepals		
	Sepals no.	3, petaloid
	Sepals colour	Two-coloured—white/pale pink (basal fl.) or pale pink/dark pink (top fl. and buds.)
	Sepals shape	Elongate-lanceolate, arched, spreading
	Sepals apex	Strongly acuminate
	Lat. sepals length	5.6–6.9(7.1)
	Lat. sepals width	0.8–1.3(1.9) (>2 × width lat. petals)
	Lateral sepal position	Spreading horizontally/slightly downwards oriented
	Median sepal length	5.9–7.0(7.4)
	Median sepal width	1.1–1.6(2.1)
	Median sepal position	Spreading, straight downwards oriented
(Lateral) Petals		
	Lat. petals no.	2, sepaloid
	Lat. petals colour	Two-coloured—white/pale pink (basal fl.) or pale pink/dark pink (top fl. and buds.)
	Lat. petals shape	Elongate-lanceolate, arched/flared, spreading
	Lat. petals apex	Strongly acuminate
	Lat. petals length	4.9–5.4(6.1)
	Lat. petals width	0.4–0.7(1.1)
	Lat. petals position	Spreading, to a subtended angle of c. 45° relative to the straight pointing median sepal
Labellum		
	Labellum type	Non-resupinate, upwards oriented
	Labellum shape	Acuminate, rhomboidal
	Labellum three-dimensionality	More-or-less planar to slightly convex
	Labellum marginal serrations	Absent
	Labellum marginal undulations	Slightly undulate
	Labellum lateral constriction	Pronounced
	Labellum median ridge	Absent
	Labellum markings	Absent
	Labellum colour	Two-coloured—white/pale pink (basal fl.) or pale pink/dark pink (top fl. and buds.)
	Labellum length	6.1–6.5(7.1) (length > 2 × width)
	Labellum width	2.2–2.8(3.1)
	Labellum base	1.5–1.7(1.9)
	Labellum dissection (lobes no.)	Shallowly 3-lobed
	Median lobe	Triangular, elongate
	Lateral lobes	Scalloped, roundish, bent upwards
	Apical part (epichile)	Flat, arched upwards, heart-shaped, flared
	Epichile length	2.3–2.5(2.8)
	Epichile width	2.2–2.8(3.1)
	Middle part (mesochile)	Tube-like (saddle-like) formed by the narrowing edges of lateral lobes
	Mesochile width	(0.1/0.2) 0.6–0.7(0.78)
	Basal part (hypochile)	Bulbous, roundish
	Hypochile width	1.5–1.7(1.9)
	Labellum adaxial epidermis	Planar cells
Spur		
	Spur shape	Saccate, spherical-ovoidal
	Spur colour	Whitish, translucent
	Spur length	2.5–2.9(3.1) (short)
	Spur diameter	1.9–2.1(2.5) (broad)
	Spur curvature	Straight
Nectar		
	Presence	Present, moderately abundant (nectar-rewarding species)
	Amount	¼–½ spur length filled
Gynostemium		
	Gynostemium type	Thick, cylindrical
	Gynostemium colour	Translucent, faintly purple pigmentated
	Gynostemium length	5.2–6.1(6.3)
	Gynostemium width	2.9–3.1(3.4)
Staminodes		
	Presence	Absent
Auricles		
	Presence	Present, laterally of the gynostemium (gynostemial auricles)
	Auricles type	Prominent
	Auricles colour	Translucent white
	Auricles texture	Verrucose
	Auricles shapes	Ovoidal to spherical
	Auricles length	0.5–0.7(1.1)
	Auricles width	0.3–0.5(0.8)
Clinandrium		
	Presence	Absent
Anther		
	Anther type	Bithecal
	Anther shape	Elongate
	Anther colour	Translucent white to yellowish
	Anther length	2.9–3.2(3.7)
	Anther width	1.2–1.5(1.8)
Thecae		
	Thecae no.	2
	Thecae position in the anther	Parallel, topmost/apical
	Thecae colour	Translucent white to yellowish
	Thecae length	3.2–3.8(4.2)
	Thecae width	1.3–1.6(1.8)
Connective		
	Presence	Present, strongly developed
	Connective colour	Intensely, purple pigmented
Anther cap		
	Presence	Absent
Pollinia		
	Pollinia type	Massulate pollinia
	Pollinia no.	2, one in each theca
	Pollinia shape	Ovoidal-elongate
	Pollinia colour	Yellow
	Pollinia length	3.1–3.5(4.1)
	Pollinia diam.	1.3–1.6(1.8)
	Contact with stigma	No stigmatic contact
Massulae		
	Massulae no.	48–60(78)
	Massulae compactness	Moderately compact
	Massulae shape	Ovoidal, roundish
	Massulae colour	Yellow
	Massulae length	0.5–0.53(0.6)
	Massulae width	0.45–0.48(0.5)
Caudicle		
	Presence	Present
	Caudicle colour	Yellow to translucent-yellow
	Caudicle length	1.2–1.6(1.4)
	Caudicle diam.	0.1–0.12
Viscidium		
	Presence	Present
	Viscidium type	Naked
	Viscidium shape	Approximately circular
	Viscidium colour	Translucent white
	Viscidium diam.	0.7–0.83(1.1)
Pollinaria		
	Pollinaria type	Massulate
	Pollinaria no.	2, one in each theca
	Pollinaria structure	Pollinia, caudicle, viscidium
	Pollinaria shape	Caudicle long (>30% pollinium length)
	Pollinaria placement on gynostemium	Proximal, parallel
Stigma		
	Stigma type	Wet
	Stigma position	Below the anther
	Stigma shape	Concave, 3-lobed, elliptic
	Stigma colour	Translucent white
	Stigma height	1.9–2.3(2.8)
	Stigma width	4.8–5.1(5.4)
	Stigmatic exudate	Present
	Stigma lobes	2 lateral, 1 median (rostellum)
Lateral lobes		
	Lat. lobes position	Lappets spreading laterally
	Lat. lobes shape	Elliptic, ovoidal, convex
	Lat. lobes colour	Translucent white
	Lat. lobes length	2.2–2.4(2.6)
	Lat. lobes diam.	1.2–1.4(1.6)
Rostellum		
	Presence	Present, above spur entrance
	Rostellum shape	Roof-like, with a prominent rostellar fold
	Rostellum colour	Translucent white
	Rostellar median fold type	Prominent
	Rostellar median fold height	2.3–2.4(2.6)
Bursicles		
	Presence	Absent
Ovary		
	Ovary type	Epigynous, unilocular
	Ovary shape	Ovoidal
	Ovary colour	Green
	Ovary length	3.1–3.3(3.6)
	Ovary width	1.6–1.8(1.9)
	Ovary resupination (torsion)	Non-resupinate
Placentation		
	Type of placentation	Parietal
Ovule		
	Type of ovule	Anatropous and tenuinucellate
	Ovule length	0.42–0.68(0.74)
	Ovule width	0.28–0.32(0.39)
Flower pedicel		
	Presence	Absent (sessile flowers)
Fruit		
	Fruit shape	Elongate, ovoidal pod
	Fruit colour	Green
	Fruit length	3.5–3.8(3.9)
	Fruit width	1.8–1.9(2.1)
	Fruiting	June–July
	Fruit set	50–70%
Seed capsule		
	Capsule shape	Ovoidal elongate
	Capsule colour	Brownish
	Capsule length	3.5–3.9(4.1)
	Capsule width	1.8–1.9(2.2)
	Capsule dehiscence/maturation	July–August
Seeds		
	Seeds shape	Elongate, ovoidal to fusiform
	Seeds colour	Brownish
	Seeds testa external ornamentation	Reticulate
	Seeds length	1.43–1.53(2.32)
	Seeds width	0.7–0.9(1.2)
	Seeds maturation	August–September/October
Embryo		
	Embryo shape	Ovoidal, roundish
	Embryo colour	Whitish-yellow
	Embryo length	0.63–0.75(1.9)
	Embryo width	0.46–0.61(0.86)
Chromosomes		
	Chromosomes no.	2n = 4x = 80 (putatively)
	Ploidy base level	x = 20 (putatively tetraploid)
Flowering time		end-June–mid-July
Reproductive strategies		
	Sexual reproduction	Allogamous
	Asexual reproduction	Vegetative propagation via root-tubers
		Apomixis/Facultatively apomictic (putatively)
Pollination		
	Pollination type	Entomophile, crossed-pollinated
	Pollination strategy	Generalised food-foraging strategy
	Pollinators	Hymenopterans (ants, bees, wasps), Dipterans (flies, mosquitos), Lepidopterans (moths, butterflies) and Coleopterans (beetles)

**Table 2:** A comprehensive, detailed list of the recorded insect species for each order is provided in the following table; several pollinators and visitors are illustrated in Figure 8, Figure 9 and Figure 10.

Insect Family	Insect Genus/Species	Pollinator Efficiency	Insect Visitor	Orchid Species
**Order Coleoptera Linnaeus, 1758 (beetles)**				
Cantharidae Imhoff, 1856 (soldier beetles)	***Cantharis obscura*** Linnaeus, 1758 (soldier beetle)	Potential/ accidental pollinator	Visitor (pollen forager)	*G. winkeliana* *D. viridis* *G. conopsea*
	***Rhagonycha lignosa*** (Müller, O.F., 1764) (golden soldier beetle)	Potential/ accidental pollinator	Visitor (pollen forager, aphids)	*G. winkeliana* *D. viridis*
Chrysomelidae Latreille, 1802 (leaf beetles)	***Altica*** Muller, 1764 (leaf beetles)	N/A	Visitor (phytophagous, fl. parts, leaves)	*G. winkeliana* *D. viridis*
Coccinellidae Latreille, 1807 (Ladybirds)	***Coccinella septempunctata*** Linnaeus, 1758 (Seven-spot Ladybird)	N/A	Visitor (carnivorous, aphids, etc.)	*G. winkeliana*
	***Hippodamia variegata*** Goeze, 1777 (variegated ladybug)	N/A	Visitor (carnivorous, aphids, etc.)	*G. winkeliana* *G. conopsea*
Elateridae Leach, 1815 (click beetles)	***Ctenicera cuprea*** (Fabricius, 1775) (common click beetle)	Accidental pollinator	Visitor (phytophagous, fl. parts, leaves)	*G. winkeliana* *P. albida* ssp. *tricuspis*
Scarabaeidae Latreille, 1802 (scarab beetles)	***Phyllopertha horticola*** (Linnaeus, 1758) (garden chafer, garden foliage beetle)	N/A	Visitor (phytophagous, fl. parts, leaves)	*G. winkeliana* *P. albida* ssp. *tricuspis* *G. conopsea*
**II. Order Diptera Linnaeus, 1758 (flies, gnats, mosquitoes)**				
Anthomyiidae Robineau-Desvoidy, 1830 (houseflies)	***Fucellia*** Robineau-Desvoidy, 1842 (seaweed flies)	Potential pollinator	Visitor (scavenger)	*G. winkeliana* *D. viridis*
	***Adia cinerella*** (Fallen, 1825) (flower fly)	Potential pollinator	Visitor (scavenger)	*G. winkeliana* *P. albida* ssp. *tricuspis*
	***Pegomya*** Robineau-Desvoidy 1830 (beet leafminer)	Potential pollinator	Visitor (scavenger)	*G. winkeliana* *D. viridis*
Calliphoridae Brauer & Bergenstamm, 1889 (survey, blow fly)	***Calliphora*** Robineau-Desvoidy, 1830 (blow fly)	Potential pollinator	Visitor (scavenger)	*G. winkeliana*
Empididae Latreille, 1804 (dagger flies)	***Empis ciliata*** Fabricius, 1787 (black dance fly)	Pollinator	Visitor (carnivorous, nectar, pollen)	*G. winkeliana*
	***Empis trigramma*** Wiedemann in Meigen, 1822 (yellow dance fly)	Pollinator	Visitor (carnivorous, nectar, pollen)	*G. winkeliana* *P. albida* ssp. *tricuspis*
Muscidae Latreille, 1802 (house flies or stable flies)	***Hydrotaea*** Robineau-Desvoidy, 1830 (house fly, dump fly)	Potential pollinator	Visitor (parasite, scavenger)	*G. winkeliana* *P. albida* ssp. *tricuspis*
	***Phaonia*** Robineau-Desvoidy, 1830 (bristleshins)	Potential pollinator	Visitor (nectar, pollen)	*G. winkeliana* *D. viridis*
	***Musca domestica*** Linnaeus, 1758 (house fly)	Potential pollinator	Visitor (parasite, scavenger)	*G. winkeliana* *D. viridis*
	***Polietes*** Rondani, 1866 (fly)	Potential pollinator	Visitor (parasite, scavenger)	*G. winkeliana*
	***Coenosia*** Meigen, 1826 (tiger flies)	Potential Pollinator	Visitor (carnivorous, nectar, pollen)	*G. winkeliana* *P. albida* ssp. *tricuspis*
	***Hebecnema*** Schnabl, 1889 (true fly)	Potential pollinator	Visitor (parasite, scavenger)	*G. winkeliana*
	***Lophosceles*** Ringdahl, 1922 (small fly)	Potential pollinator	Visitor (parasite, scavenger)	*G. winkeliana* *G. conopsea*
	***Stomoxys*** Geoffroy, 1762 (stable fly)	Potential pollinator	Visitor (parasite)	*G. winkeliana*
	***Haematobia*** Le Peletier and Serville, 1828 (true fly, horn fly)	Potential pollinator	Visitor (carnivorous)	*G. winkeliana* *G. conopses*
Scathophagidae Robineau-Desvoidy, 1830	***Scathophaga stercoraria*** (Linnaeus, 1758) (yellow dung fly)	N/A	Visitor (pollen, nectar forager)	*G. winkeliana* *D. viridis*
Sepsidae Walker, 1833 (black scavenger flies)	***Sepsis fulgens*** Meigen, 1826 (lesser dung fly)	N/A Potential pollinator	Visitor (pollen, nectar forager)	*G. winkeliana*
Syrphidae Latreille, 1802 (hoverflies, syrphids)	***Eristalis tenax*** (Linnaeus, 1758) (common drone fly)	Potential pollinator	Visitor (pollen, nectar forager)	*G. winkeliana* *D. viridis* *P. albida* ssp. *tricuspis*
	***Scaeva pyrastri*** (Linnaeus, 1758) (Pied hoverfly)	N/A Accidental pollinator	Visitor (pollen, nectar forager)	*G. winkeliana* *P. albida* ssp. *tricuspis*
	***Syrphus ribesii*** (Linnaeus, 1758) (hoverfly)	Accidental pollinator	Visitor (pollen, nectar forager)	*G. winkeliana* *D. viridis*
	***Eupeodes corollae*** (Fabricius, 1794) (common hoverfly)	Accidental pollinator	Visitor (pollen, nectar forager)	*G. winkeliana*
**III. Order Hymenoptera Linnaeus, 1758 (wasps, bees, ants)**				
Apidae Latreille, 1802 (bees, bumble bees)	***Apis mellifera*** Linnaeus, 1758 (European honey bee)	Pollinator	Visitor (pollen, nectar forager)	*G. winkeliana* *D. viridis* *P. albida* ssp. *tricuspis*
	***Bombus pratorum*** (Linnaeus, 1761), worker (early bumblebee)	Pollinator	Visitor (pollen, nectar forager)	*G. winkeliana* *G. conopsea* *P. albida* ssp. *tricuspis*
	***Nomada*** Scopoli, 1770 (nomad bees, cuckoo bees)	Potential pollinator	Visitor (pollen, nectar forager)	*G. winkeliana*
Formicidae Latreille, 1809 (ants)	***Formica fusca*** Linnaeus, 1758 (silky ant)	N/A Accidental pollinator	Visitor (carnivorous)	*G. winkeliana* *D. viridis* *G. conopsea*
	***Myrmica rubra*** (Linnaeus, 1758) (common red ant, European fire ant)	N/A Accidental pollinator	Visitor (carnivorous)	*G. winkeliana* *D. viridis* *P. albida* ssp. *tricuspis*
	***Myrmica schencki*** Viereck, 1903 (flower ant)	N/A Accidental pollinator	Visitor (carnivorous)	*G. winkeliana* *D. viridis*
**IV. Order Lepidoptera Linnaeus, 1758 (butterflies, moths)**				
Adelidae Bruand, 1851 (fairy longhorn moths)	***Adela*** Latreille, 1796 (fairy moths)	Pollinator	Visitor (nectar forager)	*G. winkeliana*
Cossidae Leach, 1815 (cossid millers or carpenter millers)	***Zeuzera pyrina*** (Linnaeus, 1761) (leopard moth)	Potential pollinator	Visitor (nectar forager)	*G. winkeliana*
Crambidae Latreille, 1810 (snout moths, grass moths)	***Crambus*** Fabricius, 1798 (snout moths)	Pollinator	Visitor (nectar forager)	*G. winkeliana* *G. conopsea* *P. albida* ssp. *tricuspis*
	***Crambus uliginosellus*** Zeller, 1850 (sod webworms)	Pollinator	Visitor (nectar forager)	*G. winkeliana* *P. albida* ssp. *tricuspis*
Erebidae (Leach, 1815) (macromoths)	***Euclidia glyphica*** (Linnaeus, 1758) (burnet companion)	Pollinator	Visitor (nectar, pollen forager)	*G. winkeliana*
Hepialidae Stephens, 1829 (ghost moths)	***Pharmacis carna*** (Denis and Schiffermüller, 1775) (ghost moths, furry moth)	Pollinator	Visitor (nectar forager)	*G. winkeliana* *G. conopsea* *P. albida* ssp. *tricuspis*
Lasiocampidae Harris, 1841 (Lappet moths)	***Dendrolimus pini*** (Linnaeus, 1758) (pine-tree lappet moth)	Pollinator	Visitor (nectar forager)	*G. winkeliana*
Nymphalidae Rafinesque, 1815 (brush-footed butterflies)	***Boloria pales*** (Denis and Schiffermüller, 1775) (shepherd’s fritillary)	Pollinator	Visitor (nectar forager)	*G. winkeliana* *G. conopsea* *P. albida* ssp. *tricuspis*
	***Erebia epiphron*** (Knoch, 1783) (small mountain ringlet)	Pollinator	Visitor (nectar forager)	*G. winkeliana*
	***Erebia medusa*** (Denis and Schiffermüller, 1775) (woodland ringlet)	Pollinator	Visitor (nectar forager)	*G. winkeliana* *P. albida* ssp. *tricuspis*
Ypsolophidae Guenée, 1845 (diamondback moths)	***Ypsolopha*** Latreille, 1796 (wainscot smudge)	Pollinator	Visitor (nectar forager)	*G. winkeliana*
Zygaenidae Latreille, 1809 (burnet or forester moths)	***Zygaena exulans*** (Reiner and Hohenwarth, 1792) (Scotch burnet)	Pollinator	Visitor (nectar forager)	*G. winkeliana* *G. conopsea*
	***Zygaena loti*** (Denis and Schiffermüller, 1775) (slender Scotch burnet)	Pollinator	Visitor (nectar forager)	*G. winkeliana* *G. conopsea*

## Data Availability

Data are contained within the article.

## Abbreviations

a.s.l.	above sea level
an-c	anther connective
an-th	anther theca
an	anther
AOO	area of occupancy
au	auricle
br	bract
BNP	Bucegi Natural Park
bur	bursicle
ca	caudicle
dhl	dehiscence lines
elev.	elevation
EOO	extent of occurrence
epi	epichile
epd	epidermis
fr	fruit
gyn	gynostemium
hyc	hypochile
la-msc	labellum mesochile
la	labellum
lat-lo	lateral labellar lobe
LCDP	Lankester Composite Dissection Plate
lpe	lateral petal
lse	lateral sepal
lsl	lateral stigmatic lobe
mas	massula/massulae
mse	median sepal
ne	nectar
ov	ovary
ov/fr-ca	ovary/fruit capsule
ovu	ovule
pa-pl	parietal placentation
pa	papilla
po	pollinia
pol	pollinarium
rof	rostellar flap
ros	rostellum
s.l.	sensu lato
s.s.	sensu stricto
sp-entr	spur entrance
sp	spur
st	stigma
sto	stomata
tfl	thecal flaps
vis	viscidium
